# FRAGTE2: An Enhanced Algorithm to Pre-Select Closely Related Genomes for Bacterial Species Demarcation

**DOI:** 10.3389/fmicb.2022.847439

**Published:** 2022-05-18

**Authors:** Jiaqi Zeng, Yuxiao Wang, Ziyao Wu, Yizhuang Zhou

**Affiliations:** ^1^Institute of Pathogeny Biology, School of Basic Medicine, Guilin Medical University, Guilin, China; ^2^Guangxi Key Laboratory of Environmental Exposomics and Entire Lifecycle Health, School of Public Health, Guilin Medical University, Guilin, China

**Keywords:** species, bacterial genome, average nucleotide identity, tetranucleotide, bacterial identification, bioinformatics, FRAGTE

## Abstract

We previously reported on FRAGTE (hereafter termed FRAGTE1), a promising algorithm for sieving (pre-selecting genome pairs for whole-genome species demarcation). However, the overall amount of pairs sieved by FRAGTE1 is still large, requiring seriously unaffordable computing cost, especially for large datasets. Here, we present FRAGTE2. Tests on simulated genomes, real genomes, and metagenome-assembled genomes revealed that (*i*) FRAGTE2 outstandingly reduces ~50–60.10% of the overall amount of pairs sieved by FRAGTE1, dramatically decreasing the computing cost required for whole-genome species demarcation afterward; (*ii*) FRAGTE2 shows superior sensitivity than FRAGTE1; (*iii)* FRAGTE2 shows higher specificity than FRAGTE1; and (*iv*) FRAGTE2 is faster than or comparable with FRAGTE1. Besides, FRAGTE2 is independent of genome completeness, the same as FRAGTE1. We therefore recommend FRAGTE2 tailored for sieving to facilitate species demarcation in prokaryotes.

## Introduction

Species demarcation is fundamental and important for both basic study and practical application in the bacterial domain. Among all methods, whole-genome approaches are considered to be the most advanced, including the widely used average nucleotide identity (ANI) (Konstantinidis and Tiedje, 2005a; Goris et al., 2007; Richter and Rosselló-Móra, 2009; Kim et al., 2014), the infrequently used average amino-acid identity (Konstantinidis and Tiedje, 2005b, 2007), FastANI (Jain et al., 2018), and the Microbial Species Identifier (MiSI) (Varghese et al., 2015), bringing species demarcation into the genome era. However, almost all these methods except for FastANI suffer from a large amount of pairwise whole-genome comparisons. In an effect to address this obstacle, we proposed a strategy called “sieving,” the process of pre-selecting closely related (intraspecies and some closely related interspecies) pairs for subsequent genome-wide alignment and calculation (Zhou et al., 2020), and developed the method called “**frag**ment **te**tranucleotide frequency correlation coefficient” (hereafter termed FRAGTE1) for it (Zhou et al., 2020). Our previous study shows that FRAGTE1 is completeness-independent, highly sensitive (~100%), and highly specific as well as with both highly reduced overall number of sieved pairs and highly improved runtime, compared with the “**tetra**nucleotide frequency correlation coefficient” (TETRA), FastANI, and the alignment-based approaches such as 16S rRNA-based approach (Zhou et al., 2020).

However, the overall amount of pairs sieved by FRAGTE1 is still large, which is computationally intensive, especially for large datasets. Taking the dataset for real genomes consisting of 61,914 queries against 5,680 references used previously as an example, a total of 2,231,656 (0.63%) pairs were sieved (Zhou et al., 2020), leaving a room for developing a more robust sieving approach. Accordingly, here we developed an enhanced version of FRAGTE (hereafter termed FRAGTE2), which is tailored for sieving. Our results showed that FRAGTE2 is more powerful than FRAGTE1 to outstandingly reduce the total amount of sieved pairs with higher sensitivity, higher specificity, and lower or comparable runtime. Thus, it is reasonable that FRAGTE2 will substitute FRAGTE1 to aid in genome-based species demarcation in future.

## Results

### The Demand for More Powerful Sieving Approach

Horizontal gene transfer may yield a high ANI of ≥96% for even distantly related species, according to Additional file 4 in Zhou et al. (2020). In this context, demarcating such species based merely on the ANI criterion may lead to incorrect conclusions. Therefore, some additional information is used to guarantee high authority. For example, the Microbial Species Identifier (MiSI) method uses ≥60% alignment fraction together with ≥96.5% ANI (Varghese et al., 2015); FastANI requires ≥50 3-kb homologous fragments between two intraspecies strains (Jain et al., 2018); Richter and Rosselló-Móra's ANI method utilizes the ANI threshold of ≥95 or 96% in combination with ≥0.99 TETRA (Richter and Rosselló-Móra, 2009). Our previous study has further demonstrated that only percentage of shared genome (PSG) rather than FRAGTE1 or TETRA can guarantee high accuracy for species demarcation, and intraspecies strains have ≥70% PSGs (Zhou et al., 2020).

**Figure 1:**
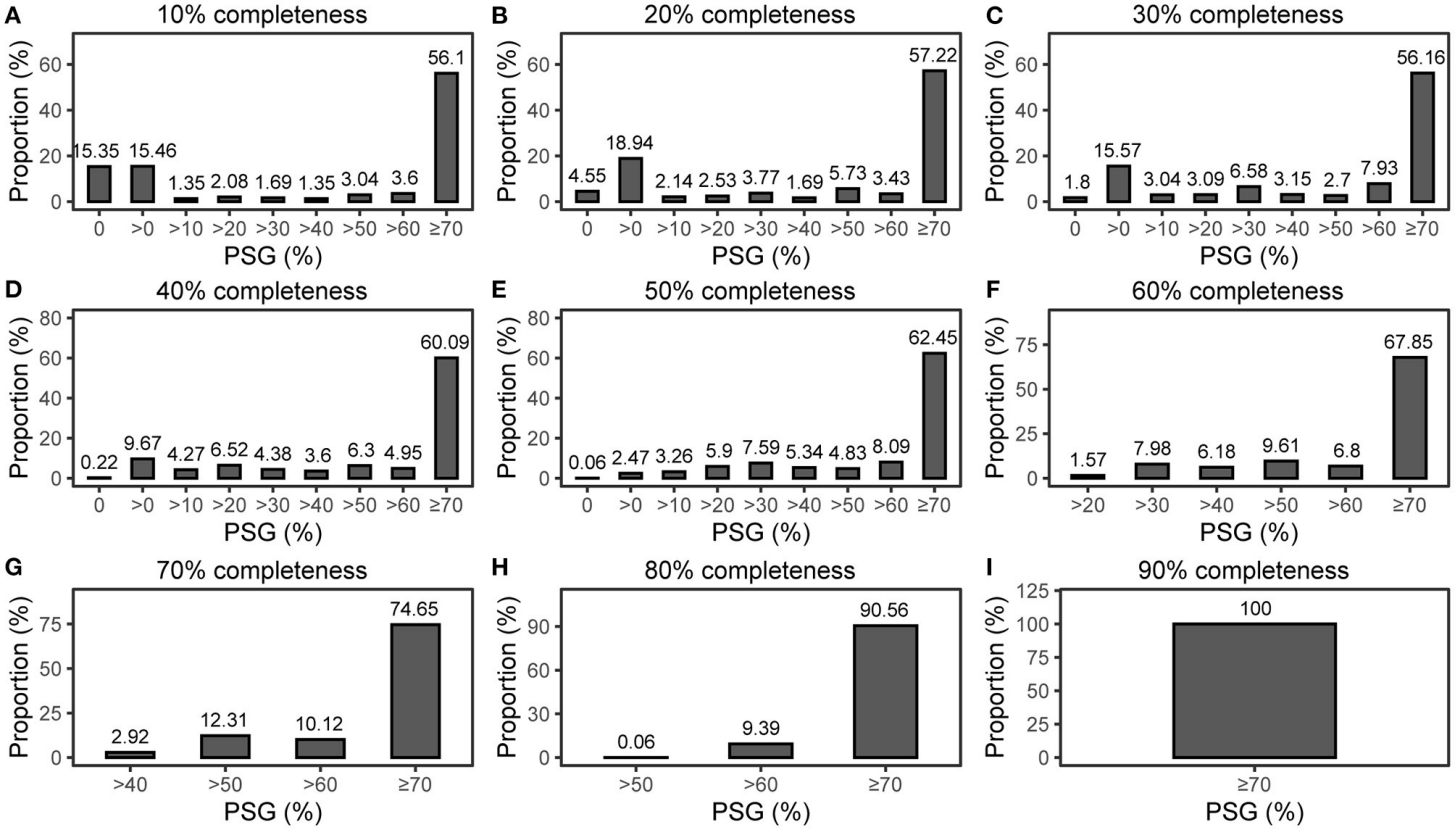
PSG distribution for intraspecific pairs. **(A–I)** for both queries and references with 10–90% completeness, respectively. All were run on 1,779 queries against 264 references with simulated 10–90% completeness in the previous study (Zhou et al., 2020).

However, our tracking found that FRAGTE1 was devised to ensure that almost all intraspecies strain pairs are sieved even when they have ~0% PSGs (Figure 1). Although intraspecies strains with <70% PSGs can be successfully sieved, they cannot be demarcated as intraspecific according to literature reports (Konstantinidis and Tiedje, 2005a; Goris et al., 2007; Richter and Rosselló-Móra, 2009; Kim et al., 2014; Varghese et al., 2015; Jain et al., 2018). Therefore, excluding such intraspecific pairs together with much more interspecific pairs may markedly reduce the overall amount of sieved pairs to decrease the computing cost for subsequent species demarcation, which is crucial, especially for large datasets. For this, we developed FRAGTE2 in this study.

### Algorithm Description

**Figure 2:**
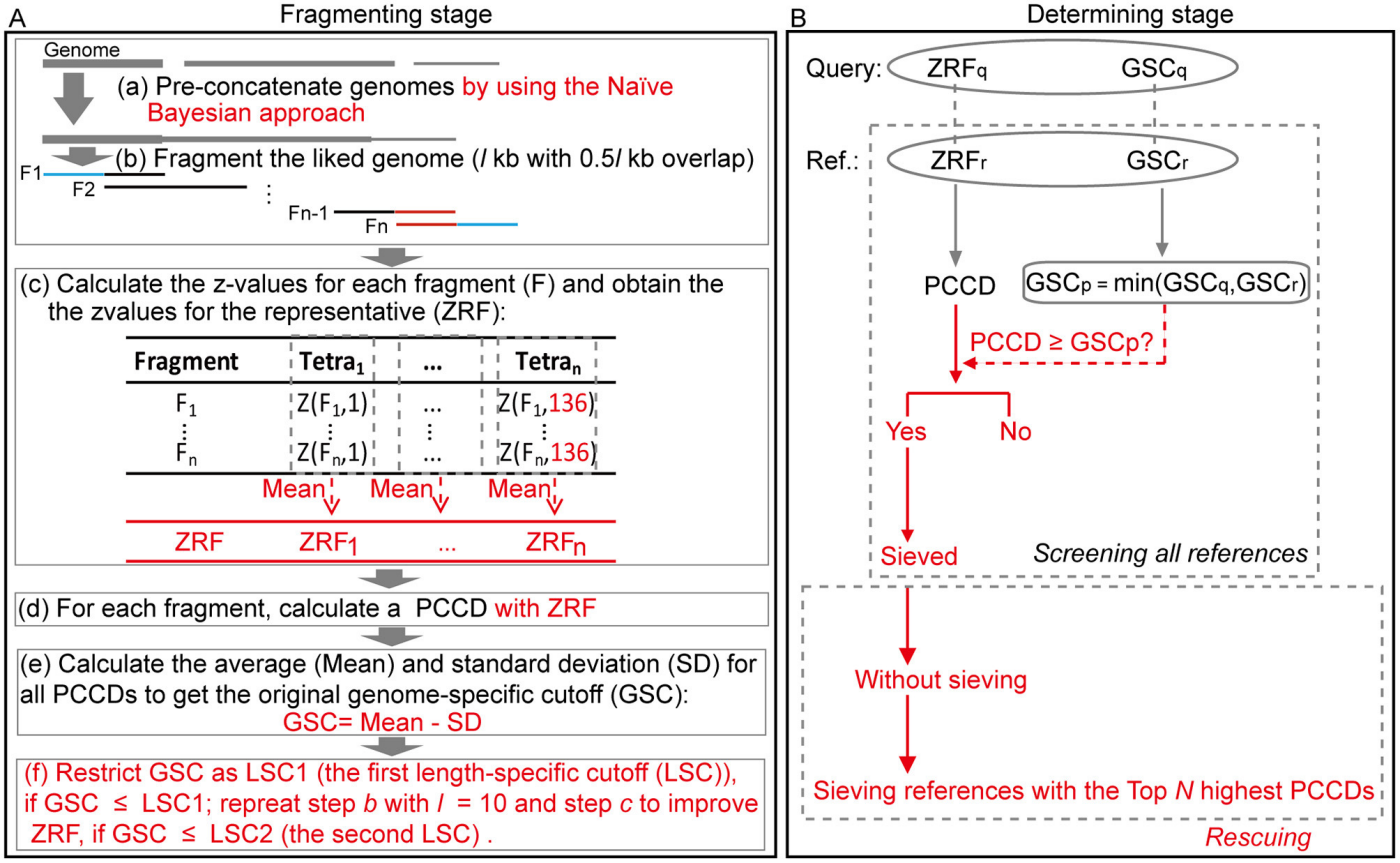
Overview of the FRAGTE2 algorithm. **(A)** Fragmenting stage. Each genome is pre-linked by using the Naïve Bayesian approach (a), and the resulting genome is separated by a sliding *l*-kb window with 0.5*l*-kb overlap (b). Subsequently, 136 z-values are generated for each fragment and then converted into the z-value for the representative (ZRF) of its genome *via* taking the average of all resultant intraspecific z-values collectively (c). In the following step, a series of Pearson's correlation coefficient distances (PCCDs) are calculated by comparing ZRF with the z-values for all fragments (d) to get a genome-specific cutoff (GSC) for their genome (e). Finally, if GSC is ≤ LSC1, restrict GSC to its corresponding LSC1; repeat step *b* with *l* = 10 and step *c* to update ZRF when GSC is ≤ LSC2. **(B)** Determining stage. An intergenomic PCCD based on ZRFs between each pair is calculated. If PCCD is ≥ GSC_p_, the GSC for the tested pair determined as the smaller between GSC for the query (GSC_q_) and for the reference (GSC_r_), this pair is sieved. In addition, in some rare conditions, if one query selects no references, references with the top *N* (100 in default) highest PCCDs are roughly sieved. Red indicates improvement compared with FRAGTE1.

FRAGTE2 uses the same workflow as FRAGTE1, consisting of fragmenting stage followed by determining stage (Figure 2). However, there are several major changes. Prior to fragmenting stage, genomes with several contigs/scaffolds are pre-concatenated based on posteriori estimates calculated by the Naïve Bayesian approach (Sandberg et al., 2001; Zhou et al., 2013), rather than pre-concatenated directly in FRAGTE1.

In fragmenting stage, FRAGTE2 first divides each (concatenated) genome by using a window of *l* kb with 0.5*l*-kb overlap (for *l* in detail, refer to Supplementary Figure S1). It is worth pointing out that only eight fragments are divided for a genome with >800 kb in FRAGTE2, when its average size of sequences is >200 kb (Supplementary Figure S1). For the benefits of such setting, please refer to the Discussion section below. Subsequently, FRAGTE2 calculates 136 z-values for tetranucleotide frequencies for each fragment. Based on the resulting z-values, FRAGTE2 calculates an average of three (for uneven number of fragments) or four (for even number of fragments) central z-values for each tetranucleotide. By this means, all resulting 136 averages are obtained and considered as the z-values for the representative fragment (ZRF). It is noteworthy that the ZRF is not from a typical fragment with maximal accumulated Pearson's correlation coefficient distance (PCCD) as described in FRAGTE1 (Zhou et al., 2020). Using this strategy, FRAGTE2 obtains more typical ZRFs than FRAGTE1 (Supplementary Figure S2), allowing FRAGTE2 to increase genome-specific cutoffs (GSCs) to achieve both higher specificity and higher sensitivity. Then, each fragment is compared with its ZRF, rather than the z-values of other non-overlapped fragments with the z-values of the selected representative fragment in FRAGTE1, to produce a PCCD. Based on all intragenomic PCCDs, FRAGTE2 calculates the average (Mean) and standard deviation to calculate a high GSC as mean minus a combination of one standard deviation and 0.01 (for details, see Materials and Methods), while FRAGTE1 calculates GSC as *mean* minus two standard deviations, due to the more typical ZRF in FRAGTE2 than in FRAGTE1 (Supplementary Figure S2). We found a fraction of genomes harbor GSCs smaller than their corresponding LSC1s (the first length-specific cutoffs (LSCs) used in this study) (Supplementary Figure S3A), which are calculated as the mean of PCCDs minus a combination of standard deviation of PCCDs and 0.01 based on the intraspecific PCCD distributions determined empirically in Zhou et al. (2020). To keep high specificity, FRAGTE2 forcedly restricts GSCs to their corresponding LSC1s when GSCs are < LSC1s. By this means, FRAGTE2 incorporates LSC1s into GSCs, without requiring comparing intergenomic PCCDs with LSC1s in determining stage, greatly reducing the runtime of FRAGTE2. It is worth pointing out that the LSC1s in FRAGTE2 are based on the size of the entire genome rather than divided fragments in FRAGTE1 (Supplementary Figure S3A), which greatly improve specificity to filter more interspecific pairs (Supplementary Figure S3B). Further, we found that some GSCs are even lower than their corresponding LSC2s, the second LSCs devised in FRAGTE2, which are calculated as the mean of PCCDs minus 2 standard deviations of PCCDs (Supplementary Figure S4) based on the intraspecific PCCD distributions determined in Zhou et al. (2020), indicating that the tetranucleotides within such genomes are heterogeneous and ZRFs obtained by FRAGTE2 are poorly representative for other intragenomic fragments. Under this condition, FRAGTE2 conducts a second fragmenting by setting *l* to a smaller 10 kb to obtain a more typical ZRF for each of such genomes, ensuring that these genomes can be successfully sieved by FRAGTE2 without losing high specificity meanwhile.

In determining stage, FRAGTE2 calculates an intergenomic PCCD between a pair of genomes based on their ZRFs. Then, FRAGTE2 compares the PCCD with the GSC for this pair (termed GSC_p_, the smaller one of the two GSCs for a given pair). If PCCD is ≥GSC_p_, this pair is ultimately sieved. After screening all available references, a query without any satisfied references is then subject to rescuing. The references with the top *N* (100 in default) highest PCCDs are roughly sieved based on the assumption that the tetranucleotide composition from the interspecific fragments is much more similar than interspecific fragments (Teeling et al., 2004; Dick et al., 2009; Laczny et al., 2015; Zhou et al., 2019, 2020). By this strategy, such query may also obtain its targets to avoid missing its species demarcation. However, it is worth pointing out that this strategy cannot ensure that all intraspecific targets are sieved, mainly due to the sequencing errors by sequencers including Ion Torrent Personal Genome Machine (PGM) and 454 GS-FLX (for details, see Discussion below).

### Improved Sieving Performance on Simulated Genomes

**Figure 3:**
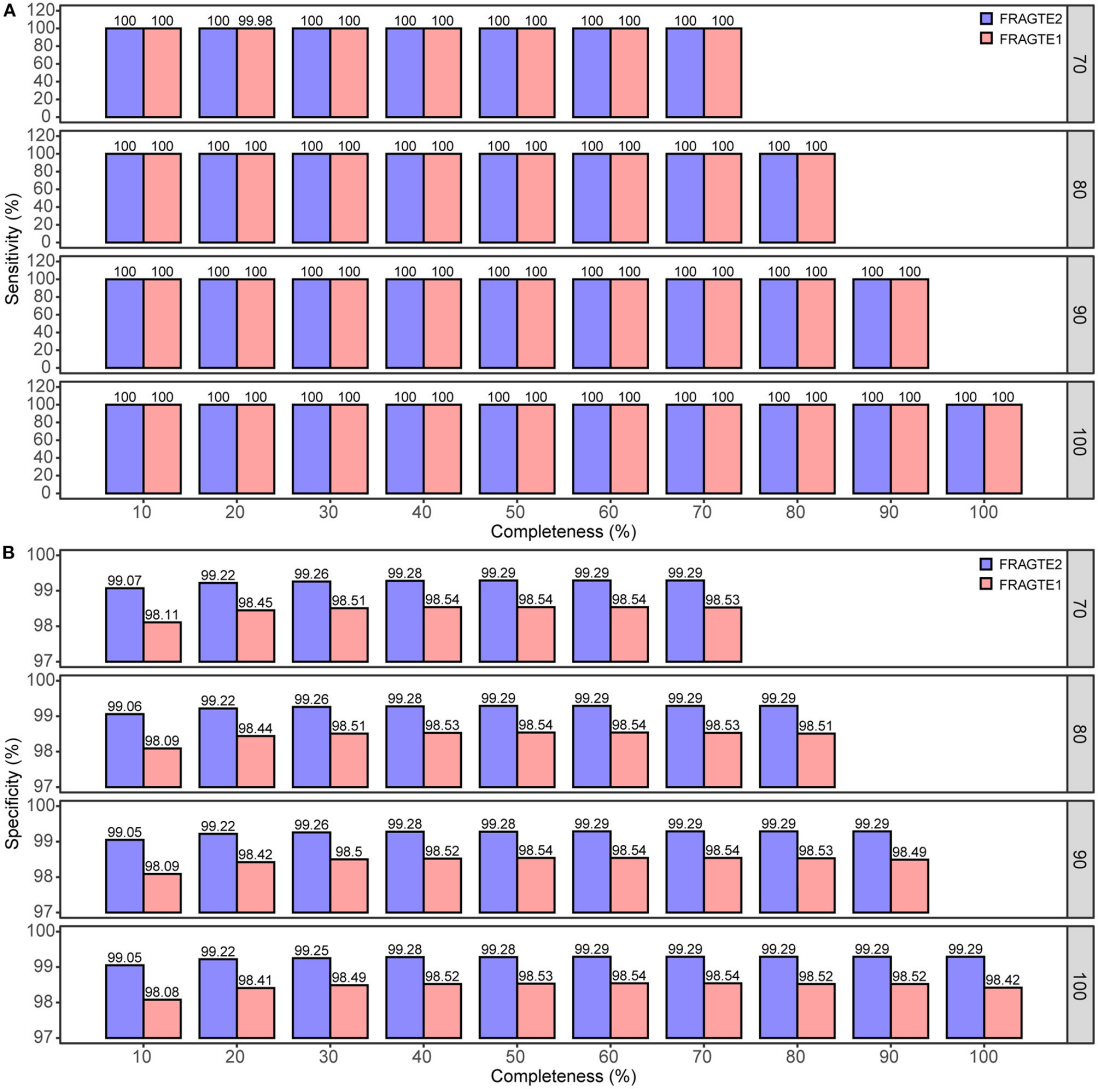
Comparison of sieving sensitivity and specificity of FRAGTE2 and FRAGTE1 on simulated genomes. **(A)**, for sensitivity; **(B)**, for specificity. The boxed number on the right represents 70–100% of PSG.

**Figure 4:**
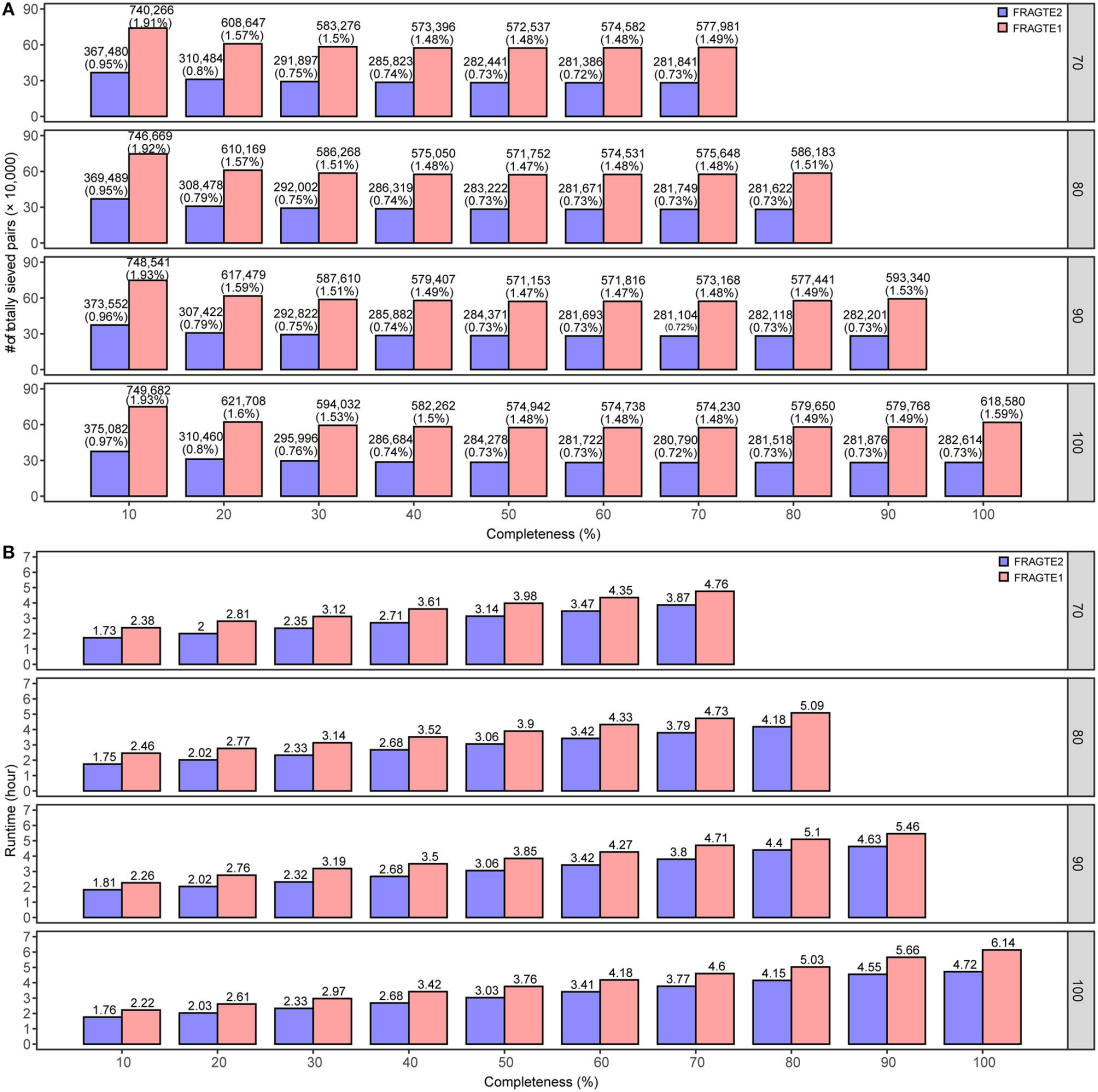
Comparison of overall number of sieved pairs and sieving runtime between FRAGTE2 and FRAGTE1 on simulated genomes. **(A)**, for overall number of sieved pairs; **(B)**, for sieving runtime. The boxed number on the right represents 70–100% of PSG.

A total of 6,230 complete genomes comprising 38,812,900 pairs were applied to investigate the sieving improvement by FRAGTE2 on simulated genomes. We generated simulated genomes *via* extracting each genome twice with 70–100% of PSGs to form a intraspecific genome pair. This strategy ensured that all intraspecific pairs were with 70–100% PSGs. Then, the pairs from different genomes were putatively considered as interspecific, as they were not guaranteed to have 70–100% PSGs, although some are indeed intraspecific. In this way, we simulated 10–70%, 10–80%, 10–90%, and 10–100% completeness for genomes with 70%, 80%, 90%, and 100% of PSGs, respectively. Our results showed that FRAGTE2 strikingly yielded perfect sensitivities of 100% for all simulations (Figure 3A), indicating that FRAGTE2 is also completeness-independent. However, FRAGTE1 only achieved a ~99.98% of sensitivity for genomes with 20% of completeness and 70% of PSGs. Our tracking revealed that the pair from the genome GCA_001723525.1 (*Lactobacillus salivarius*) fail to pass their LSC (Supplementary Table S1), demonstrating the outperformance of using the averaged z-values in FRAGTE2 instead of the z-values with the largest accumulated PCCD in FRAGTE1 as ZRF. For specificity, FRAGTE2 greatly filtered approximately 99.05–99.29% of interspecies pairs (Figure 3B), whereas FRAGTE only correctly filtered about 98.08–98.54% of interspecies pairs, showing that FRAGTE2 has higher specificity than FRAGTE1. Taken together, FRAGTE2 sieved pairs with both higher sensitivity and higher specificity than FRAGTE1. Accordingly, FRAGTE2 greatly reduced ~50% of the total number of sieved pairs from 571,153–749,682 (~1.47–1.93% of total pairs) by FRAGTE1 to 280,790–376,378 (~0.72–0.97% of total pairs) (Figure 4A), which will dramatically save the runtime for subsequent genome-wide alignments and calculations of species demarcation. Finally, we compared the runtimes of both versions and found that FRAGTE2 run faster than FRAGTE1 (Figure 4B). Accordingly, FRAGTE2 lowered the executive time for both the processes of sieving as well as alignment and calculation after sieving due to decreased amount of totally sieved pairs, which together improved the run efficiency for genome-wide species demarcation. In summary, all these findings validated that FRAGTE2 is superior to FRAGTE1.

### Improved Sieving Performance on Real Genomes

**Figure 5:**
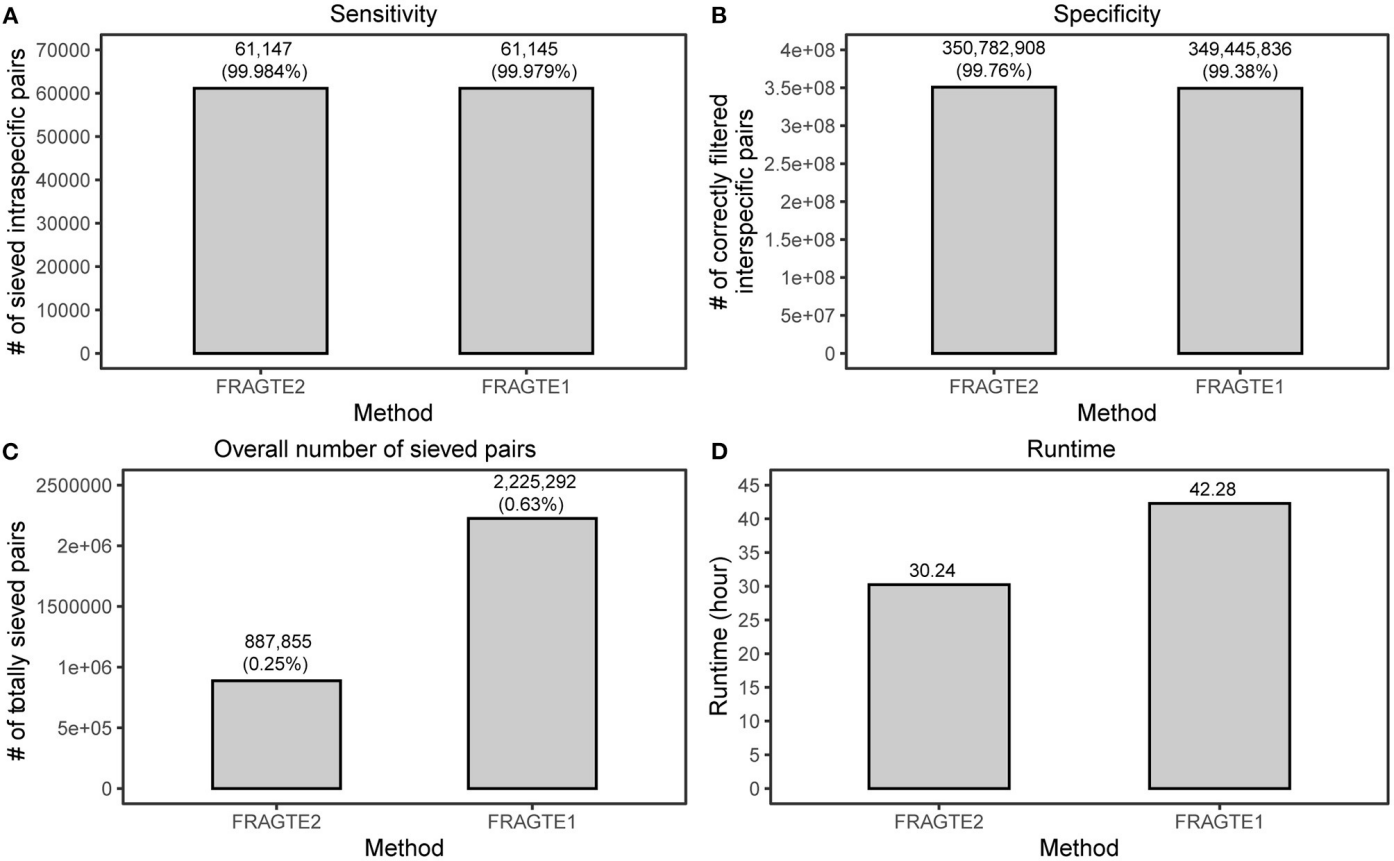
Improved sieving performance on real genomes. **(A)**, for sensitivity, the sensitivity of FRAGTE2 is calculated as (4 + 61143)/(4 + 61143 + 10), while the sensitivity of FRAGTE1 is calculated as (61143 + 2) /(61143 + 2+13); for details, refer to Supplementary Figure S5 and Supplementary Tables S7, S8. **(B)**, for specificity. **(C)**, for overall number of sieved pairs. **(D)**, for runtime.

A large database of real genomes containing 61,914 queries and 5,680 references, as used previously (Zhou et al., 2020), was further employed to compare sieving performance between FRAGTE2 and FRAGTE1 on real genomes. This database encompasses 61,914-labeled intra- and 351,609,606 interspecies pairs. Then, four metrics including sensitivity, specificity, the amount of totally sieved pairs, and runtimes were compared between the two versions, respectively. For sensitivity, a total of 61,143 intraspecific pairs were captured by both FRAGTE2 and FRAGTE1 (Supplementary Figure S5A). Besides, FRAGTE2 uniquely sieved 23-labeled intraspecific pairs, among which four were truly intraspecies pairs with ≥95% ANI and ≥70% PSG (Supplementary Tables S2, S3). In contrast, FRAGTE1 uniquely outputted 388-labeled intraspecific pairs. However, only two of them were truly intraspecific pairs (Supplementary Tables S4, S5). These findings imply that FRAGTE2 is more specific than FRAGTE1. Then, we checked the labeled intraspecific pairs unsieved by FRAGTE2 or FRAGTE1 and found that only 14 of 757 were with ≥95% ANI and ≥70% PSG for FRAGTE2 (Supplementary Figure S5B and Supplementary Table S6). We further found that five of 14 queries obtained their other intraspecific references sieved by FRAGTE2 with four having more probabilities (higher ANIs or PSGs) (Supplementary Table S7), meaning that only ten were truly missed by FRAGTE2. One possible reason is due to the sequencing errors by sequencers including Ion PGM and 454 GS-FLX (for details, see Discussion below). In contrast, a larger fraction (16/392) of labeled intraspecific pairs with ≥95% ANI and ≥70% PSG were unsieved by FRAGTE1 (Supplementary Figure S5C and Supplementary Table S8). Further analysis exhibited that only four obtained their other intraspecific references sieved by FRAGTE1, with three having an intraspecies target with more probabilities (Supplementary Table S9). The remaining 13 queries were considered to be substantially unsieved by FRAGTE1. So, FRAGTE2 achieved a higher sensitivity of ~99.984% than that of ~99.979% by FRAGTE1 (Figure 5A). As 58,120 (93.87%) queries and 4,335 (76.32%) references are incomplete (Zhou et al., 2020), this finding demonstrated that FRAGTE2 is also independent of genome completeness.

Despite higher sensitivity, FRAGTE2 filtered more interspecific pairs than FRAGTE1 (Figure 5B), supporting that FRAGTE2 is more specific than FRAGTE1. Furthermore, we found that FRAGTE2 dramatically decreased the overall amount of sieved pairs, approximating only 39.90% of totally sieved pairs of FRAGTE1 (Figure 5C). This is a distinguished advantage for FRAGTE2, which will outstandingly reduce the computing cost for subsequent genome-based species demarcation. Finally, we compared the runtimes and found that FRAGTE2 run faster than FRAGTE1, saving about 12 h (Figure 5D). Together with the reduced overall amount of sieved pairs, FRAGTE2 dramatically reduces the total executive time for species demarcation. To sum up, all these outcomings demonstrated that FRAGTE2 has superiority over FRAGTE1 on real genomes.

### Improved Sieving Performance on Metagenome-Assembled Genomes (MAGs)

**Figure 6:**
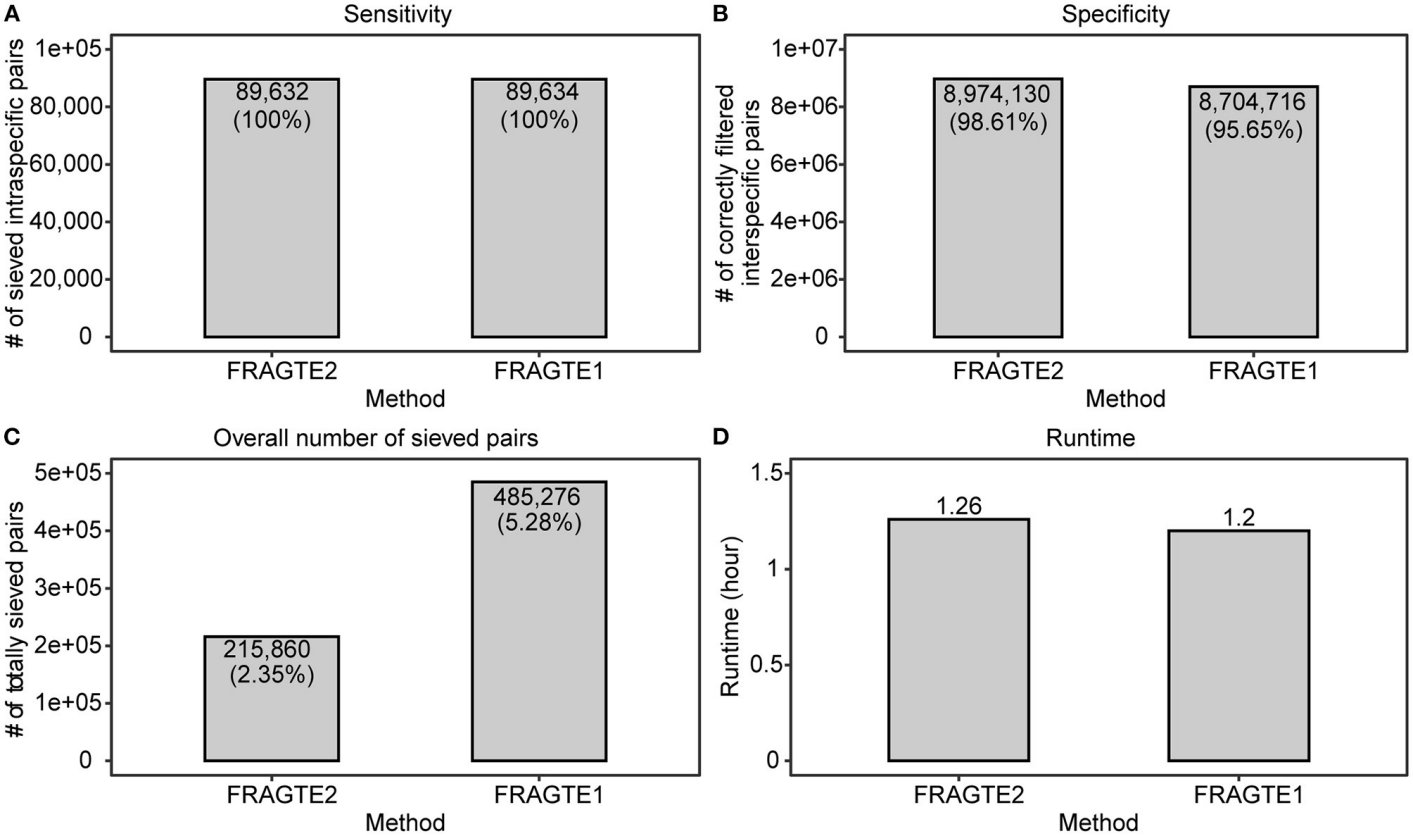
Improved sieving performance on metagenome-assembled genomes (MAGs). **(A)**, for sensitivity; **(B)**, for specificity; **(C)**, for overall number of sieved pairs; **(D)**, for runtime.

A previously used set of 3,032 MAGs (Zhou et al., 2020) were used as both queries and references to demonstrate the sieving improvement of FRAGTE2 on MAGs. After excluding pairs between identical MAGs, the resulting dataset contains 89,634 intraspecific pairs with ≥70% PSGs and 9,100,358 interspecific pairs. Our results showed that both versions achieved ~100% of sensitivity (Figure 6A), with only one exception between GCA_900282665.1 and GCA_900285595.1 for FRAGTE2. However, both these MAGs successfully obtained their more probable targets with higher PSGs (Supplementary Table S10), meaning that they will still be correctly demarcated. In this context, FRAGTE2 also achieved a perfect sensitivity of 100%. As the vast majority of MAGs are incomplete (Zhou et al., 2020), this finding demonstrates that FRAGTE2 is also completeness-independent. Moreover, this finding indicated that FRAGTE2 is even able to filter less probable intraspecific pairs, which is very useful for consequent species demarcation, supporting the high specificity of FRAGTE2. Despite achieving identical sensitivity, FRAGTE2 filtered more interspecies pairs than FRAGTE1 (Figure 6B), further supporting that FRAGTE2 is more specific than FRAGTE1. Accordingly, FRAGTE2 reduced ~55.52% (269,416) of overall sieved pairs than FRAGTE1 (Figure 6C), markedly reducing the computing cost required for subsequent species demarcation. Finally, we compared their runtimes and found that FRAGTE2 required a little more runtime than FRAGTE1 (Figure 6D). One possible reason is that 25% (758/3032) MAGs are with highly heterogeneous tetranucleotides to produce GSCs lower than their corresponding LSC2s and thus require the second fragmenting with 10-kb window (Figure 2). Our result confirmed that FRAGTE2 run faster than FRAGTE1 (Supplementary Figure S6), without second fragmenting. However, the increased runtime of FRAGTE2 was dramatically slight (~0.06 h), compared with the increased runtime (~154.12 h) suffering from the augmented pairs sieved by FRAGTE1 for species demarcation afterward (Supplementary Figure S7). Accordingly, FRAGTE2 is efficient to amazingly reduce runtime for species demarcation in aggregate. In a conclusion, all the above outcomings demonstrated that FRAGTE2 is superior to FRAGTE1 on MAGs.

## Materials and Methods

### Datasets Used in This Study

A dataset of 6,230 complete genomes, which was used for selection of 1,779 queries and 264 references in the previous study (Zhou et al., 2020), was applied to evaluate FRAGTE2 performance on simulated genomes. Besides, the same datasets of real genomes and MAGs, as utilized in the previous study (Zhou et al., 2020), were employed to compare sieving performance on real genomes and MAGs, respectively. All are deposited in https://github.com/Yizhuangzhou/FRAGTE2.

### Calculation of ANI and PSG

Pairs were aligned genome-wide using the NUCmer tool (version 4.00) (Marcais et al., 2018), with default parameters except for “–L 1000.” Then, we used the “.delta” file to calculate ANI and PSG. Each aligned stretch between two sequences has a unique header. The header line lists seven values, the start and end in the reference, the start and end in the query, the number of errors (non-identities +indels), the number of similarity errors (non-positive match scores), and stop codon, followed by a string of signed digits, one per line before the next header equaling zero. One negative digit means a gap (deletion) in the reference. The length of each alignment was calculated as the aligned stretch length in the reference added with the number of negative digits; the number of identical nucleotides was calculated as the aligned stretch length subtracted by the number of errors. On the basis of the reference genome, ANI was then calculated as follows:


(1) $$\begin{array}{c} ANI\;\text{=}\\ \frac{\sum \text{(the aligned stretch length + the number of negative digits - the number of errors})}{\sum \text{(the aligned stretch length + the number of negative digits)}} \end{array}$$

Some stretches, such as paralogs and other repeats, overlapped in the alignment, which would introduce biases in the PSG. Thus, the PSG for a pair was calculated as follows:


(2) $$\begin{array}{c} PSG\;=\\ \frac{\sum \text{Aligned positions for query+}\sum \text{Aligned positions for reference}}{L1+L2} \end{array}$$

Where the numerator was calculated by counting all aligned positions (in terms of base pairs) in both genomes of a pair to eliminate the effect of overlapping alignments, and the denominators *L*_1_ and *L*_2_ were the total length of the query and reference (also in terms of base pairs), respectively.

### Pre-Processing Genomes

Within each genome, sequences with size <1 kb are discarded. Then, the average size of all resultant sequences is calculated and recorded for subsequent FRAGTE2 sieving. Subsequently, draft genomes with multiple sequences are pre-concatenated by using the Naïve Bayesian approach, since a fraction of sequences are short (<10 kb) and the Naïve Bayesian classifier is more suitable for short sequences than TETRA-like methods (Sandberg et al., 2001). In this study, a posteriori probability for each sequence with size ≥1 kb is calculated on the basis of the trained tetranucleotides of its whole genome. Subsequently, sequences are sorted based on the resulting posteriori probabilities and accordingly concatenated.

### Fragmenting Stage

In fragmenting stage, FRAGTE2 divides each (processed) genome into several fragments to get a ZRF and GSC, including several steps detailed below.

#### Dividing Genomes

The processed genomes with size ≥10 kb are remained for dividing. Given that the total length of a processed genome was denoted *L*, the genome is divided according to Supplementary Figure S1. If *L* is <40 kb, no dividing is performed and the entire genome is selected as the representative fragment; if *L* is within the range of 40 to 800 kb, the genome is fragmented into 8 fragments by setting *l* to *L*/4; if *L* > 800 kb, *l* is set to *L*/4 when the average size of this genome is >200 kb, which is different from that in FRAGTE1, or otherwise set to 200 kb, which is identical to that in FRAGTE1.

#### ZRF Calculation

After genome fragmenting, 136 z-values are calculated for each fragment according to the previous methods (Teeling et al., 2004; Zhou et al., 2019, 2020). In this way, all fragments obtain their 136 z-values and a set of the z-values are obtained for each tetranucleotide within each genome. Then, for each tetranucleotide, an average of the z-values is obtained *via* averaging the three (for odd number of fragments) or four (for even number of fragments) central z-values. Finally, all 136 averages are collectively considered as ZRF for this genome.

#### GSC Calculation

Based on ZRF and their z-values, a set of PCCDs are calculated for each genome according to the previous methods (Teeling et al., 2004; Zhou et al., 2019, 2020). Then, the average (*Mean*) and standard deviation (*SD*) of all resulting PCCDs are calculated and used to compute a GSC for its genome as follows:


(3) $$\begin{array}{l} GSC=M\text{ean}-SD-0.01 \end{array}$$

Where 0.01 is for boundary effect.

It is noteworthy that GSC calculation in FRAGTE2 is different from that in FRAGTE1, which is calculated as follows (Zhou et al., 2020):


(4) $$\begin{array}{l} GSC=M\text{ean}-2\text{*}SD \end{array}$$

Therefore, GSC in FRAGTE2 is larger than that in FRAGTE1 to improve specificity.

#### Restricting GSC on the Basis of LSC1

GSC is restricted according to its LSC1. In this study, LSC1 is calculated as follows:


(5) $$\begin{array}{l} LSC1=\;Mean_{intra,kb}-SD_{intra,kb}-0.01 \end{array}$$

Where *kb* is the size of the entire genome rather than the length of divided fragments in FRAGTE1 and is set to 200 when *kb* >200; 0.01 is for boundary effect.

To keep high specificity, GSC is forcedly set to LSC1 when GSC is < LSC1.

#### Improving ZRF With 10-Kb Fragmenting

A second LSC termed LSC2 is devised in this study. LSC2 is calculated as follows:


(6) $$\begin{array}{l} LSC2=\;Mean_{intra,kb}-2\text{*}SD_{intra,kb} \end{array}$$

Where *kb* is the size of the entire genome rather than the length of divided fragments in FRAGTE1 and is set to 200 when *kb* >200.

Low GSCs (< LSC2s) indicate that tetranucleotides within genomes are heterogeneous. The ZRFs selected in FRAGTE2 may be poorly representative for other fragments, which may lead to unsieving for these genomes. To yield more typical ZRFs, a second fragmenting with *l* set to 10 kb is performed and then an improved ZRF is yielded for each of such genomes (details to see above).

### Determining Stage

For a given pair, FRAGTE2 obtains two GSCs: one for query (termed GSC_q_) and the other for reference (termed GSC_r_). The smaller one between the two GSCs is considered as the GSC of this pair (termed GSC_p_). Based on ZRFs, a PCCD can be computed according to the previous studies (Teeling et al., 2004; Zhou et al., 2019, 2020). If the PCCD is ≥GSC_p_, the pair is sieved.

After screening all available references, queries without any satisfied references are subject to rescuing. The references with the top *N* (100 in default) highest PCCDs are roughly sieved for each of such queries.

### Implementation of FRAGTE2

The FRAGTE2 algorithm is mainly implemented in Perl (v5.16.3). To accelerate running, the Perl package “Statistics::R,” which is the module to control R interpreter and interacts with embedded R programs, is applied to perform statistics such as calculating ZRF and PCCDs by using R. This strategy greatly reduces the runtime of FRAGTE2.

### Runtime Assessment

In this study, all runtimes were evaluated by using a single compute node with two Intel® Xeon® Silver 4114 20-core processors with serial execution (single thread, single process).

## Discussion

### The Benefits of Improved Setting *l* in FRAGTE2

In FRAGTE2, we have one improvement for setting *l* (Supplementary Figure S1), namely that only eight fragments are divided for the genome with >800 kb when its average size of sequences is >200 kb. By contrast, *l* is forcedly set to 200 kb for such genome, regardless of its average size in FRAGTE1. Taking simulated dataset with 100% of PSGs and 100% of completeness as an example, our experiment shows that this setting greatly increases GSCs (Supplementary Figure S8). Further analysis revealed that this setting does not reduce any sieving sensitivity (Supplementary Figure S9A), but improves sieving specificity (Supplementary Figure S9B) and reduces the overall amount of sieved pairs (Supplementary Figure S9C) and sieving runtime (Supplementary Figure S9D), demonstrating the advantages of such setting in FRAGTE2.

### The Reasons for High Efficiency of FRAGTE2

Although a second fragmenting is additionally required for genomes with low GSCs, FRAGTE2 still is faster than or comparable with FRAGTE1. Collectively, at least the following four designs account for it. First, FRAGTE2 uses embedded R programs rather than the pure Perl programs as in FRAGTE1 to perform statistics such as calculating ZRFs and PCCDs, greatly improving sieving efficiency. Second, when the average size of a given genome is >200kb, only eight fragments are divided for such genome even with >800 kb in FRAGTE2 (Supplementary Figure S1), benefitting from the superiority of ZRF in FRAGTE2 over that in FRAGTE1. This setting reduces the amount of divided fragments and thus accelerates efficiency of FRAGTE2 (Supplementary Figure S9D). Third, LSCs are incorporated into GSCs in FRAGTE2, without requiring comparing intergenomic PCCDs with LSCs in determining stage, thereby greatly reducing its runtime. Finally, only 136 tetranucleotides are utilized after discarding reverse complementary ones in FRAGTE2, whereas all 256 of them are used in FRAGTE1, which reduces ~46.88% of runtime.

### Deleterious Effect of Sequencing Errors on Sieving Performance

The previous study has shown that Ion PGM and 454 GS-FLX have more sequencing errors than the comparable Illumina MiSeq, especially for homopolymer stretches (Liu et al., 2012; Loman et al., 2012). Our checking shows that all 14 truly intraspecific pairs unsieved by FRAGTE2 (Supplementary Table S6) have at least one genome sequenced by Ion PGM for each (Supplementary Table S11). Accordingly, we further explored whether their ZRF differences have really resulted from these sequencing-generated homopolymer-associated indels. Expectedly, we found that all 14 harbor such indels (Supplementary Table S11). Take the pair between GCA_001315865.1 and GCA_000970205.1 as an example, we found that the dominant different ZRFs between them are for the tetranucleotide GGGG (Supplementary Figure S10), implying that Ion Torrent PGM generated sequencing errors for regions with poly[d(G)] or poly[d(C)]. The remaining tetranucleotides with apparently different z-values contain two or three continuous Gs or Cs within them, which may be also affected by the less accuracy of the stretches containing poly[d(G)] or poly[d(C)], as their calculation relies on their component trinucleotide GGG or CCC and dinucleotide GG or CC according to the TETRA approach (Teeling et al., 2004; Zhou et al., 2019, 2020). Besides, Ion Torrent PGM also showed a steadily decreasing accuracy across the read (Loman et al., 2012), which may also account for other tetranucleotides with inapparent z-value differences. In this context, sequencing errors may pose deleterious effect on FRAGTE2 unsieving these 14 pairs, indicating that the genome quality is prerequisite for sieving. However, on the contrary, this finding demonstrates that FRAGTE2 is greatly specific to even exclude genomes with low quality for avoiding incorrect species demarcation. Nevertheless, it is worth stressing that Illumina sequencing is more popular than Ion PGM sequencing and 454 GS-FLX sequencing at present and, in most conditions, requires no special precautions.

### Species Concept and Species Demarcation in Prokaryotes

So far, species concepts for eukaryotes have not been successfully applied to demarcate species for prokaryotes (Ward, 1998). For example, the eukaryotic biological species concept, depending on interbreeding, is not applicable to prokaryotes because they are asexual (Ward, 1998); the evolutionary species concept, which defines a species as a lineage (an ancestral-descendant sequence of populations) evolving separately from others and with its own evolutionary role and tendencies, is difficult to directly demarcate species for prokaryotes due to the reasons of unknown phylogenetic relationships, taxonomic rank arbitrary, or chimeric history (Doolittle and Zhaxybayeva, 2009). Therefore, demarcating species for prokaryotes has not been conceptually driven, but progressed historically through empirical improvements in parallel with technical developments (Rosselló-Mora and Amann, 2001), and species demarcation does not really reflect the natural speciation (species concept) of prokaryotes.

Early species-demarcation approaches mainly relied on phenotypic characteristics. However, they are fraught with problems, especially the subjectivity of selecting certain phenotypic characteristics (Ward, 1998). To circumvent such problems, taxonomists united around a consensus demarcating species primarily based on overall genotypic similarity and then phenotypic differences for fine-scale differentiation (Doolittle and Zhaxybayeva, 2009). As a result, most of the currently used approaches are based on genotypic similarity, measured as evolutionary distance for a single gene, several housekeeping genes, or even the whole genome. For example, the most widely used approach with a single gene is based on the evolutionary distances between 16S rRNA genes using a routinely applied 97% (Tindall et al., 2010) or a more stringent threshold of 98.65% (Kim et al., 2014); approaches based on several genes include the species identification tool (Mende et al., 2013), multilocus sequence typing (Maiden et al., 1998), and multilocus sequence analysis (Thompson et al., 2005); and whole genome-based approaches include DNA–DNA hybridization (DDH) (Wayne et al., 1987), average amino-acid identity (Konstantinidis and Tiedje, 2005b, 2007), ANI (Konstantinidis and Tiedje, 2005a; Goris et al., 2007; Richter and Rosselló-Móra, 2009; Kim et al., 2014), FastANI (Jain et al., 2018), and MiSI (Varghese et al., 2015). Among these approaches, the genome-based approaches are considered to be most powerful, because they are more reliable and unbiased and have higher resolution than those using a single or multiple genes.

### Significance of FRAGTE2

FRAGTE2 developed in this study is tailored specially to sieve genome pairs for whole-genome species demarcation afterward. Strikingly, *via* excluding more interspecific pairs, intraspecific pairs with <70% of PSGs, and even less probable pairs with ≥70% of PSGs, FRAGTE2 outstandingly reduces the genome pairs required for species demarcation due to its high specificity. Besides, FRAGTE2 is more sensitive and highly or comparably improves computational efficiency for sieving than FRAGTE1. So, FRAGTE2 can replace FRAGTE1 to assist genome-based species-demarcation approaches, including ANI (Konstantinidis and Tiedje, 2005a; Goris et al., 2007; Richter and Rosselló-Móra, 2009; Kim et al., 2014), average amino-acid identity (Konstantinidis and Tiedje, 2005b, 2007), and MiSI (Varghese et al., 2015), or even some multiple-gene-based approaches such as the species identification tool using 40 marker genes (Mende et al., 2013). FRAGTE2 is especially indispensable for large databases of hundreds of thousands of pairs. Since species demarcation is fundamental for both basic study and practical application, it is reasonable that FRAGTE2 will also be important and widely used in bacterial field. Besides, it is worth pointing out that FRAGTE2 may be applicable for single-cell sequencing assembled genomes in addition to genomes and MAGs tested in this study. We hope that FRAGTE2 will facilitate bacterial and archaeal studies examining species and their contributions to the environment in future.

## Data Availability Statement

The code and accession numbers presented in this study are deposited in the Github repository (https://github.com/Yizhuangzhou/FRAGTE2).

## Author Contributions

JZ performed the most of analysis. YW and ZW performed the remaining analysis. YZ directed this project, wrote the manuscript, and get the fundings for this project. All authors contributed to the article and approved the submitted version.

## Funding

This work was jointly supported by the Natural Science Foundation of China (No. 32060144), Guangxi Research Foundation for Science & Technology Base and Talent Special (No. AD19110054), the Natural Science Foundation of Guangxi (No. 2020GXNSFAA159032), and the grant from Guangxi Key Laboratory of Molecular Medicine in Liver Injury and Repair (No. GXLIRMMKL-201910) for YZ.

## Conflict of Interest

The authors declare that the research was conducted in the absence of any commercial or financial relationships that could be construed as a potential conflict of interest.

## Publisher's Note

All claims expressed in this article are solely those of the authors and do not necessarily represent those of their affiliated organizations, or those of the publisher, the editors and the reviewers. Any product that may be evaluated in this article, or claim that may be made by its manufacturer, is not guaranteed or endorsed by the publisher.
