# Supplementary material for: FRAGTE2: An Enhanced Algorithm to Pre-Select Closely Related Genomes for Bacterial Species Demarcation
Source: Front Microbiol. 2022 May 18;13:847439. doi: 10.3389/fmicb.2022.847439 (PMC9158502; doi:10.3389/fmicb.2022.847439)
Supplement: Supplementary file 1 [file Data_Sheet_1.PDF]

Supplementary Material

A

```
# L in base pair (bp)
if (L < 40,000) {
    # short genomes
    kb = int (L/10,000)*10;
    GSC = LSCkb;
    # LSCkb: LSC when size is kb
}
else { # long genomes
    l = L / 4;
    if (l > 200,000) {
     if (Avg<200,000){
     # Avg: the average size of all
     sequences within genome
       l = 200,000;
    }
}
```

B

L (kb)	*l* (kb)	# of fragments	GSC
[10,40)	NA	NA	LSC1
[40,800]	*L* /4	8	GSC
>800			
Average <200	200	≥8	GSC
Average ≥200	*L* /4	8	GSC

Supplementary Figure 1. Setting of *l* in FRAGTE2. A, the pseudo code for setting *l*; B, summary when *L* is different. *L*, the length of a genome (kb); NA, not applicable, represents no fragmenting; GSC, genome-specific cutoff; LSG1, length-specific cutoff 1; red, modification compared with FRAGTE1.

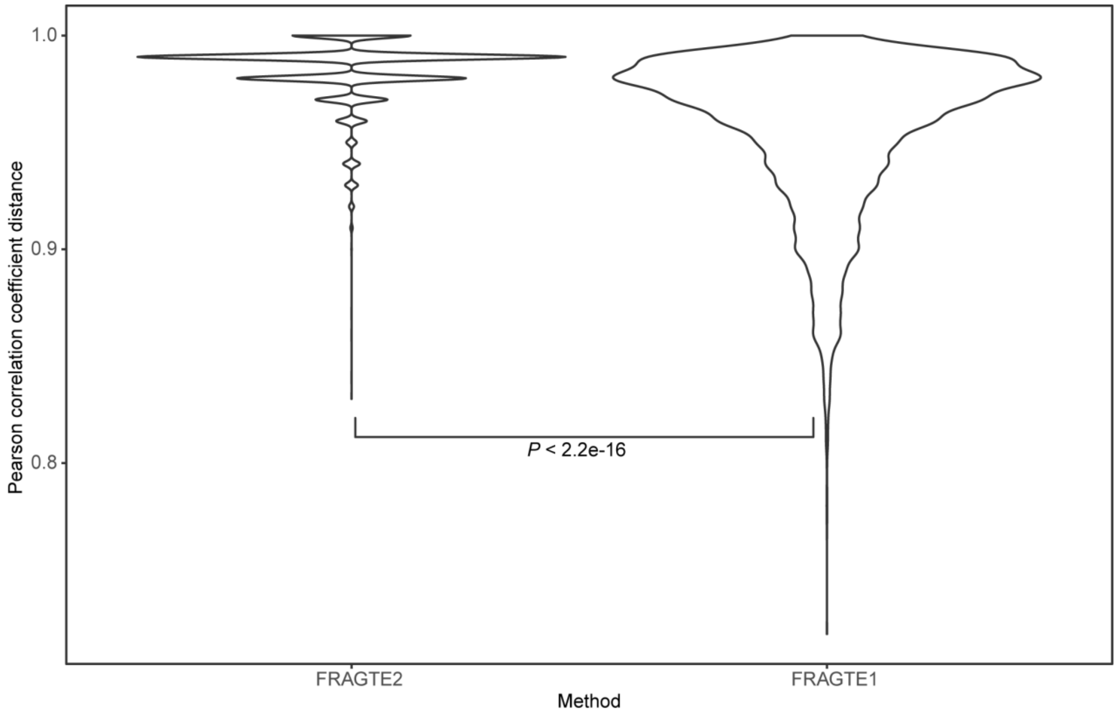

Supplementary Figure 2. Distribution of Pearson correlation coefficient distance between intraspecific pairs by using ZRFs in FRAGTE2 or FRAGTE1. The example shown here is from 6230 simulated genomes with 10% of completeness and 70% of PSGs; *P*-value, paired Wilcoxon signed rank test.

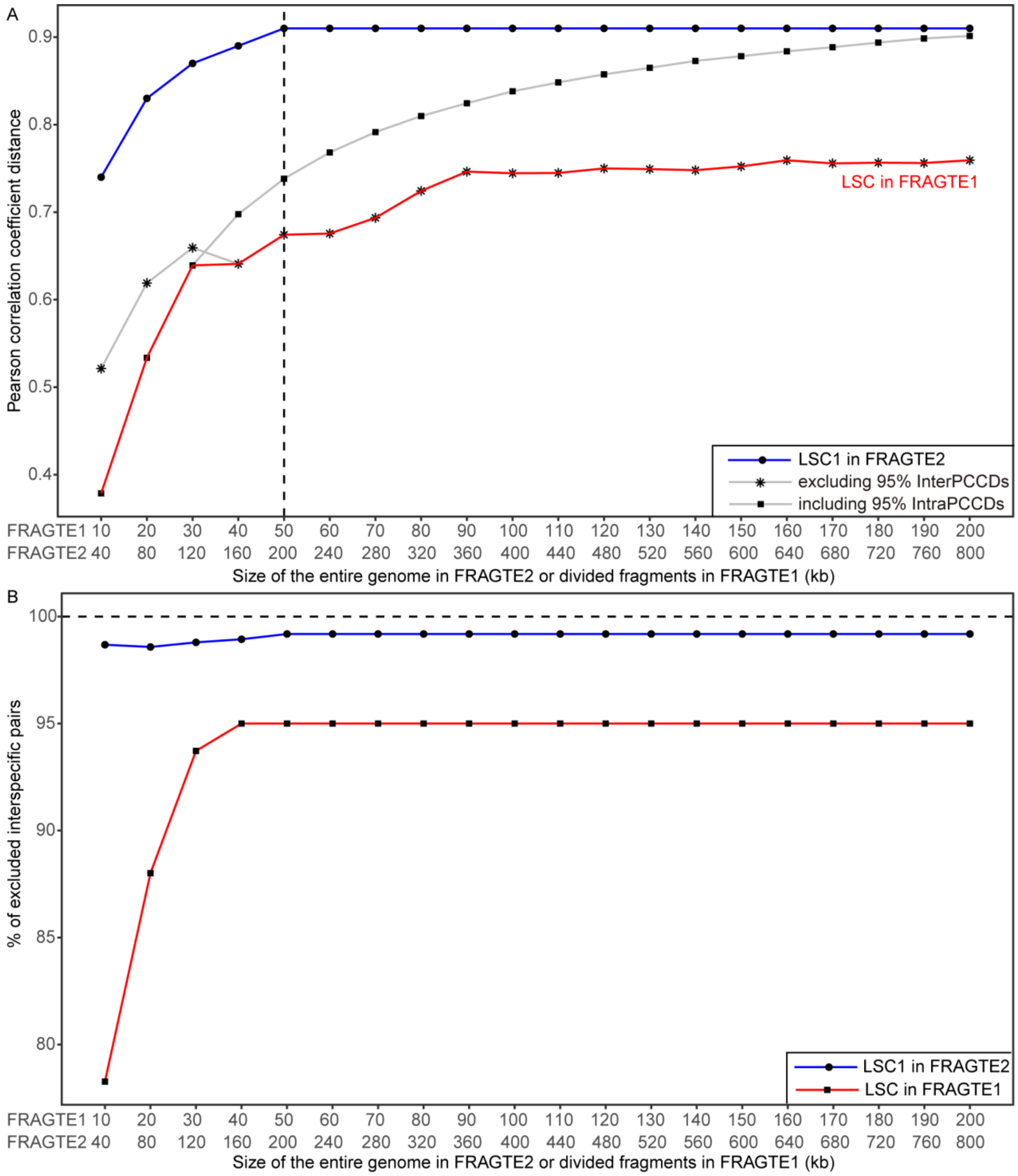

Supplementary Figure 3. Exhibition and sensitivity of the first length-specific cutoffs (LSC1s) in FRAGTE2 or LSCs in FRAGTE1. A, exhibition of the LSC1s. InterPCCDs, interspecific PCCDs; IntraPCCDs, intraspecific PCCDs. B, specificity to exclude interspecific pairs. All were based on empirically-determined PCCD distributions (Fig. 3B and Additional file 2: Table S1 in (Zhou et al., 2020)). For the size of entire genome is >200 kb, the corresponding LSC1 is according to PCCD distribution of 200 kb, as the maximal size in Fig. 3B and Additional file 2: Table S1 in (Zhou et al., 2020) is 200 kb.

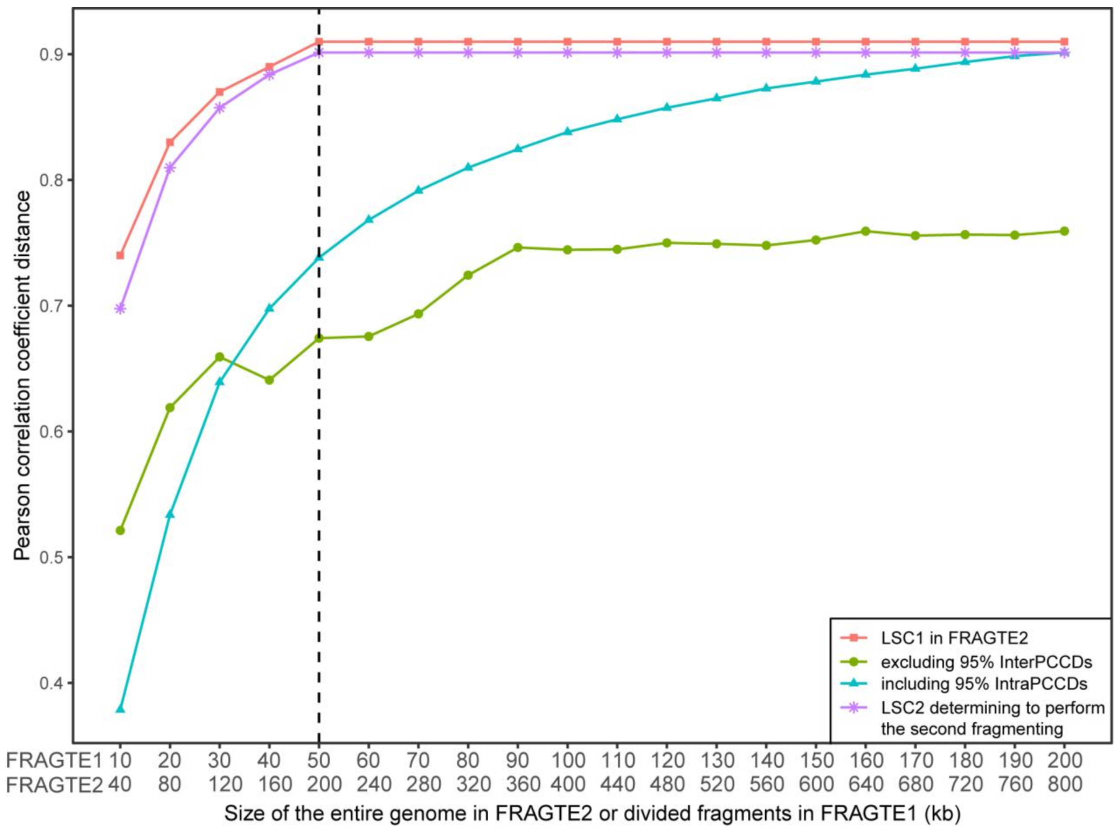

Supplementary Figure 4. Showing of the second length-specific cutoffs (LSC2s) in FRAGTE2. InterPCCDs, interspecific PCCDs; IntraPCCDs, intraspecific PCCDs. All were based on empirically-determined PCCD distributions (Fig. 3B and Additional file 2: Table S1 in (Zhou et al., 2020)). For the size of entire genome is >200 kb, the corresponding LSC is according to PCCD distributions of 200 kb, as the maximal size in Fig. 3B and Additional file 2: Table S1 in (Zhou et al., 2020) is 200 kb.

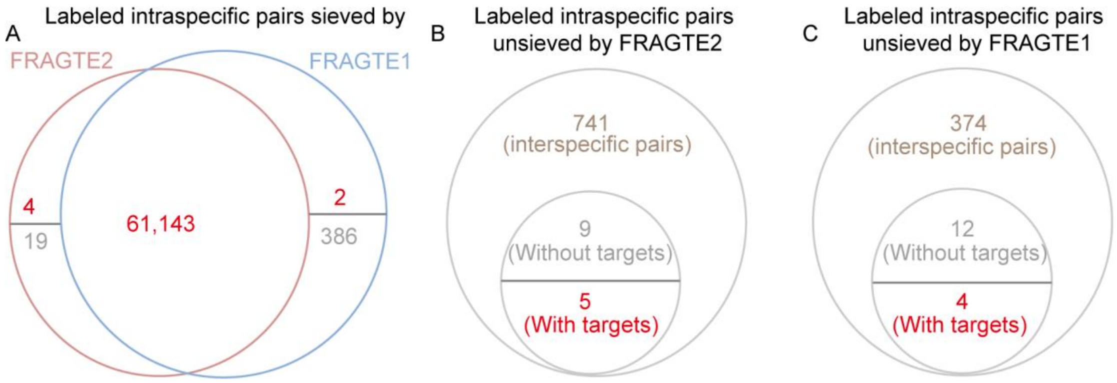

Supplementary Figure 5. Intraspecific pairs sieved or unsieved by FRAGTE2 or FRAGTE1. **(A)**, Venn diagram for labeled intraspecific pairs sieved by FRAGTE2 and FRAGTE1. red, labeled intraspecific pairs sieved by both versions or truely intraspecific pairs uniquely sieved by each version; gray, interspecific pairs. **(B)**, Venn diagram for labeled intraspecific paris unsieved by FRAGTE2. **(C)**, Venn diagram for labeled intraspecific paris unsieved by FRAGTE1.

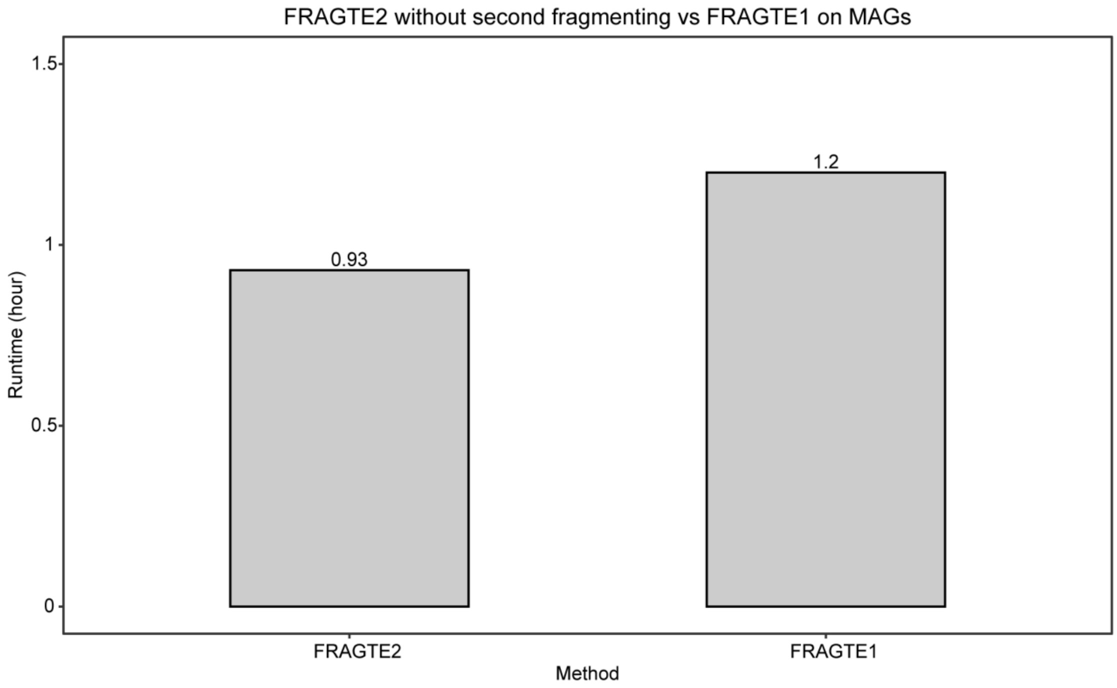

Supplementary Figure 6. Runtime comparision between FRAGTE2 without second fragmenting and FRAGTE1 on MAGs.

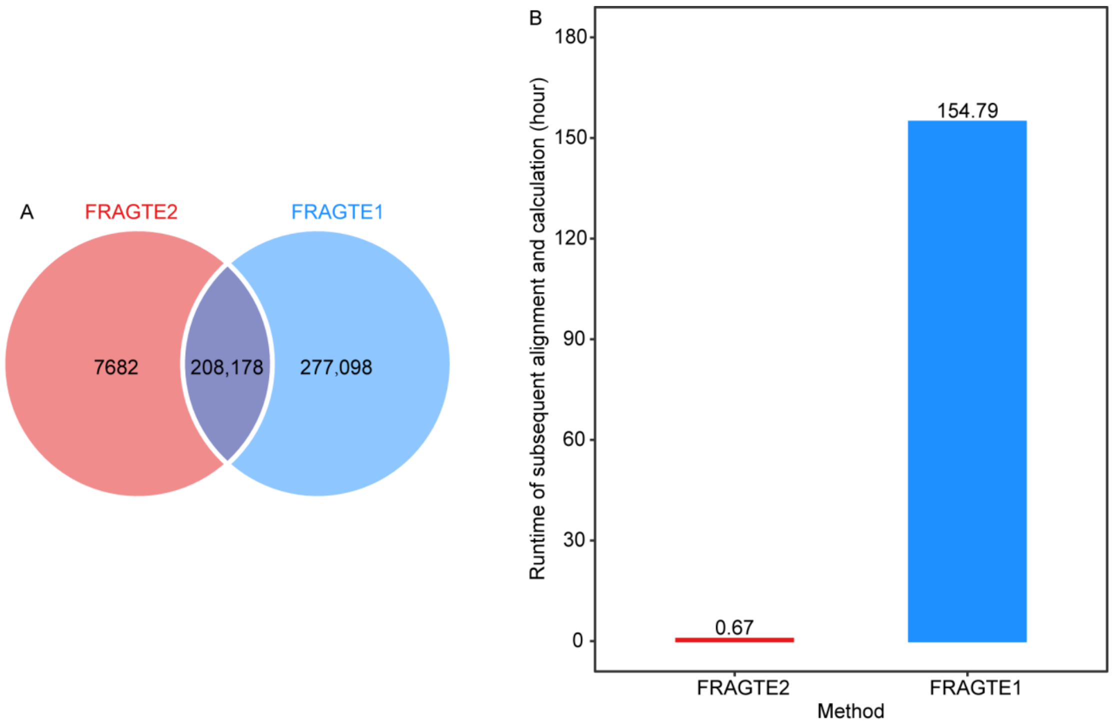

Supplementary Figure 7. Runtime comparision between pairs sieved by FRAGTE2 and pairs sieved by FRAGTE1 for subsequent species demarcation on MAGs. **(A)**, two-way Venn diagram for the overall pairs sieved by FRAGTE2 and FRAGTE1. The absolute numbers of the shared and unique pairs sieved by the two versions are shown. **(B)**, the runtime for subsequent genome-wide alignment and calculation for the unique pairs sieved by each version.

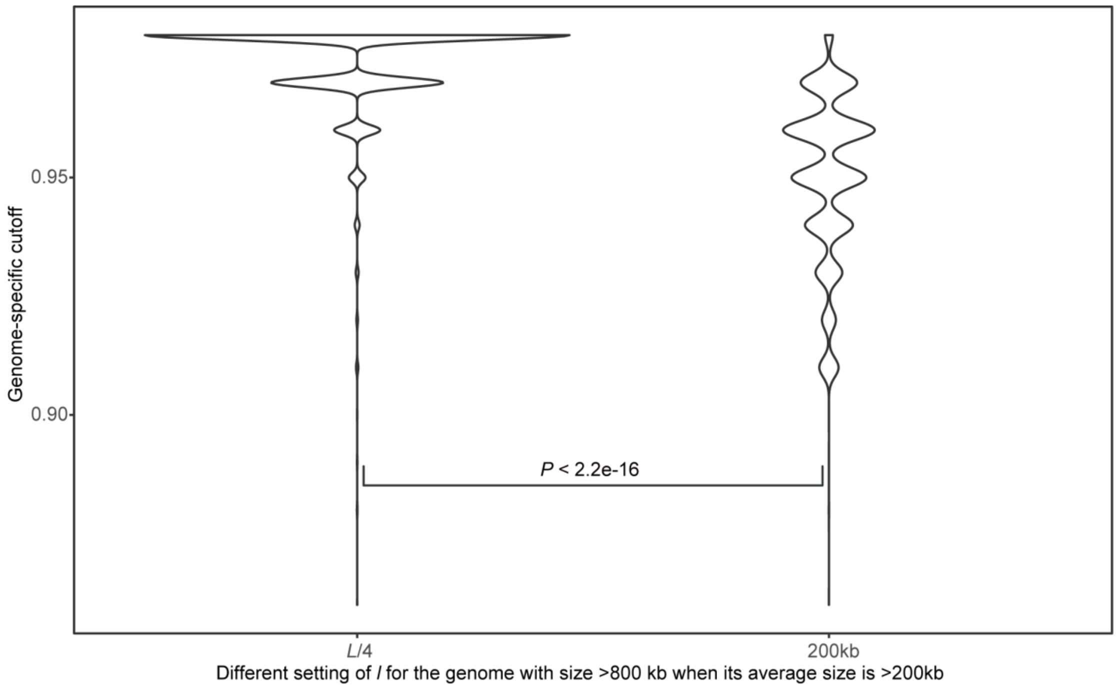

Supplementary Figure 8. GSC comparision between FRAGTE2 with and withoug the improved setting *l*. *P*-value, paired Wilcoxon signed rank test. The example showed here is based on the simulated dataset with 100% of PSGs and 100% of completeness.

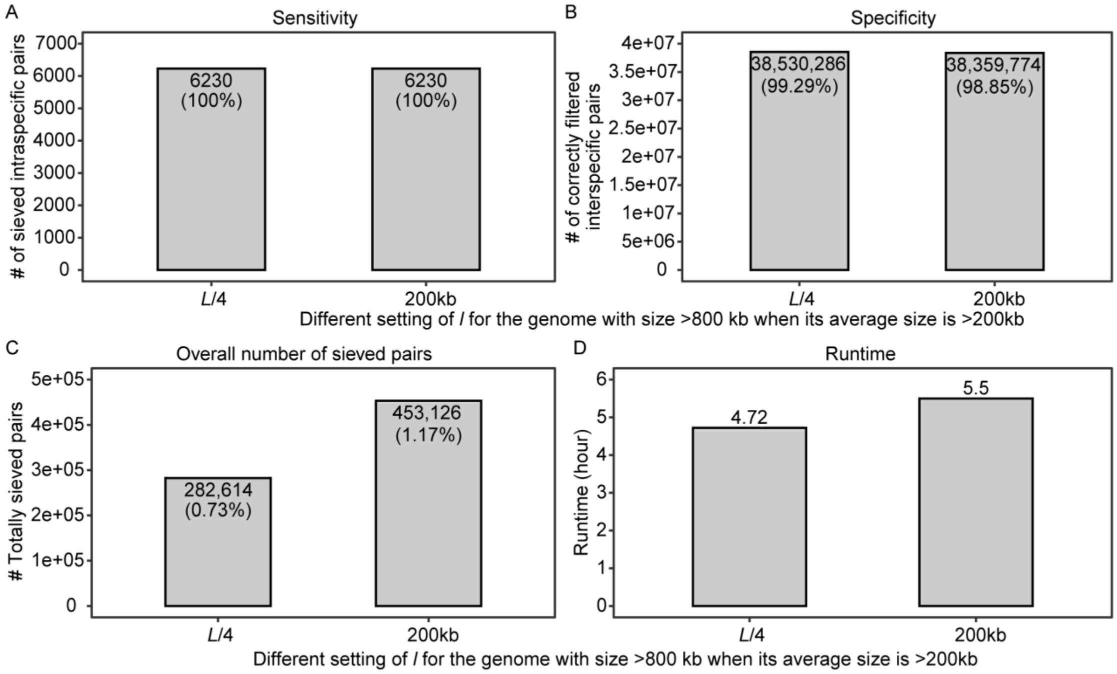

Supplementary Figure 9. Sieving performance between FRAGTE2 with and withoug the improved setting *l*. *L*, the total size of a genome. For detalis, see Fig. S1. The example showed here is based on the simulated dataset with 100% of PSGs and 100% of completeness.

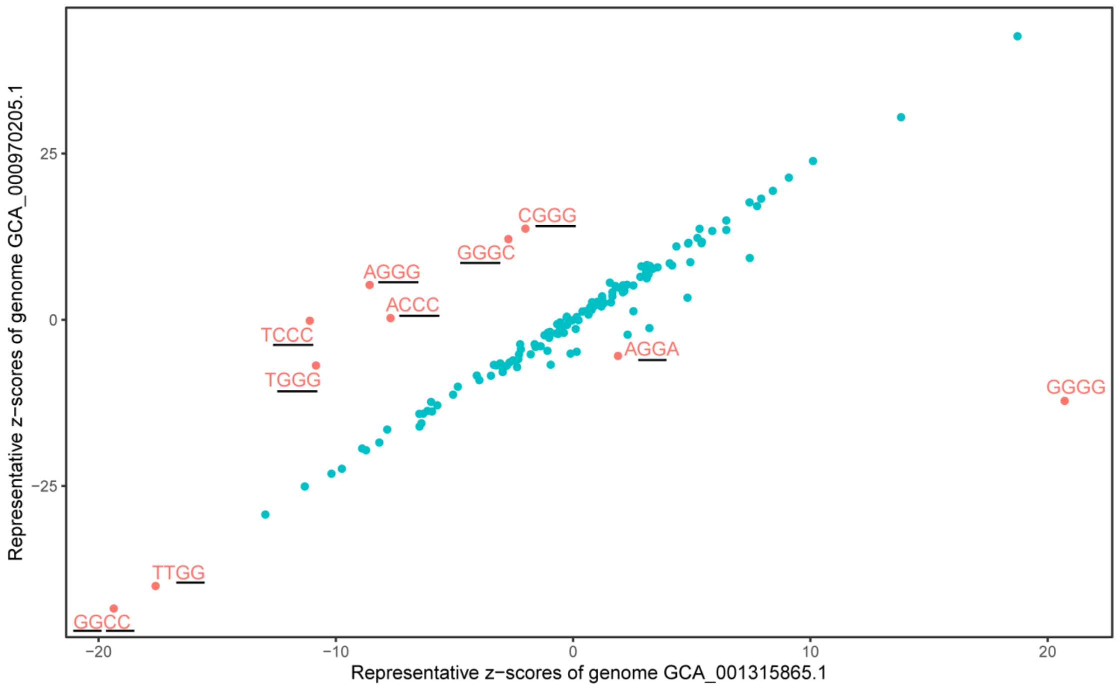

Supplementary Figure 10. Apparently different TNFs between genomes GCA_001315865.1 and GCA_000970205.1. Highlighted in pink, tetrenuclotide with apparently different z-score.

Supplementary Table 1. FRAGTE1 misses its sieving on the simulated genome pair from the genome GCA_001723525.1 (*Lactobacillus salivarius*) with 20% of completeness and 70% of PSG. P1, PCCD based on ZRFs; P2, LSC, length-specific cutoff in FRAGTE1. For details, please see the FRAGTE1 study (Zhou et al., 2020).

**Query**	**Query name**	**Reference**	**Reference name**	**P1**	**LSC**
GCA_001723525.1	*Lactobacillus salivarius*	GCA_001723525.1	*Lactobacillus salivarius*	0.70	0.74

Supplementary Table 2. The true intraspecific pairs from labeled intraspecific pairs uniquely sieved by FRAGTE2 on real genomes. PCCD, Pearson correlation coefficient distance; GSC_q, genome-specific cutoff for query; GSC_r, genome-specific cutoff for query; ANI, average nucleotide identity; PSG, percentage of shared genome.

**Query**	**Query name**	**Reference**	**Reference name**	**PCCD**	**GSC_q**	**GSC_r**	**ANI (%)**	**PSG (%)**
GCA_000472165.1	*Ochrobactrum intermedium*	GCA_000182645.1	*Ochrobactrum intermedium*	0.90	0.96	0.98	99.22	95.32
GCA_001312025.1	*Lysinibacillus boronitolerans*	GCA_000772935.1	*Lysinibacillus boronitolerans*	0.92	0.96	0.94	99.58	99.65
GCA_000319595.1	*Cronobacter sakazakii*	GCA_000982825.1	*Cronobacter sakazakii*	0.91	0.98	0.98	96.20	82.23
GCA_001436555.1	*Lactobacillus senioris*	GCA_001312065.1	*Lactobacillus senioris*	0.90	0.95	0.96	99.47	99.97

Supplementary Table 3. The indeed interspecific pairs from labeled intraspecific pairs uniquely sieved by FRAGTE2 on real genomes. PCCD, Pearson correlation coefficient distance; GSC_q, genome-specific cutoff for query; GSC_r, genome-specific cutoff for query; ANI, average nucleotide identity; PSG, percentage of shared genome.

Query	Query name	Reference	Reference name	PCCD	GSC_q	GSC_r	ANI (%)	PSG (%)
GCA_001061665.1	*Rothia mucilaginosa*	GCA_000175615.1	*Rothia mucilaginosa*	0.9	0.96	0.97	90.41	58.38
GCA_001065135.1	*Rothia mucilaginosa*	GCA_000175615.1	*Rothia mucilaginosa*	0.9	0.96	0.97	90.48	58.99
GCA_900108415.1	*Selenomonas ruminantium*	GCA_000424065.1	*Selenomonas ruminantium*	0.9	0.96	0.96	84.2	4.87
GCA_900109435.1	*Ruminococcus flavefaciens*	GCA_000518765.1	*Ruminococcus flavefaciens*	0.91	0.96	0.96	85.79	7.08
GCA_000702545.1	*Selenomonas ruminantium*	GCA_000424065.1	*Selenomonas ruminantium*	0.9	0.98	0.96	83.33	4.06
GCA_900111255.1	*Olsenella umbonata*	GCA_900105025.1	*Olsenella umbonata*	0.91	0.96	0.98	84.61	12.13
GCA_000191725.2	*Anaerococcus prevotii*	GCA_000024105.1	*Anaerococcus prevotii*	0.91	0.93	0.97	85.91	22.76
GCA_001708285.1	*Leifsonia xyli*	GCA_001647635.1	*Leifsonia xyli*	0.91	0.96	0.98	85.09	22.14
GCA_000725195.1	*Snodgrassella alvi*	GCA_000600005.1	*Snodgrassella alvi*	0.9	0.95	0.97	88.67	8.41
GCA_001278935.1	*Leifsonia xyli*	GCA_001647635.1	*Leifsonia xyli*	0.91	0.97	0.98	86.01	10.72
GCA_001690705.1	*Gilliamella apicola*	GCA_000599985.1	*Gilliamella apicola*	0.9	0.98	0.97	83.8	17.44
GCA_001458255.1	*Thalassobius mediterraneus*	GCA_001458435.1	*Thalassobius mediterraneus*	0.91	0.94	0.95	85.86	29.25
GCA_000007725.1	*Buchnera aphidicola*	GCA_000521565.1	*Buchnera aphidicola*	0.9	0.95	0.97	89.82	0.95
GCA_000799165.1	*Methylotenera versatilis*	GCA_000093025.1	*Methylotenera versatilis*	0.93	0.98	0.98	83.19	3.18
GCA_000384375.1	*Methylotenera versatilis*	GCA_000093025.1	*Methylotenera versatilis*	0.93	0.98	0.98	83.99	3.1
GCA_001648115.1	*Buchnera aphidicola*	GCA_000521565.1	*Buchnera aphidicola*	0.9	0.95	0.97	86.67	1.33
GCA_000007665.1	*Leifsonia xyli*	GCA_001647635.1	*Leifsonia xyli*	0.92	0.98	0.98	85.11	21.89
GCA_001065485.1	*Rothia mucilaginosa*	GCA_000175615.1	*Rothia mucilaginosa*	0.9	0.96	0.97	90.52	58.14
GCA_000826125.1	*Bhargavaea cecembensis*	GCA_000348905.1	*Bhargavaea cecembensis*	0.91	0.96	0.95	83.91	12.65

Supplementary Table 4. The true intraspecific pairs from labeled intraspecific pairs uniquely sieved by FRAGTE1 on real genomes. P1, PCCD based on ZRFs; P2, PCCD based on z-scores of the fourfold longer fragment; LSC, length-specific cutoff in FRAGTE1. For details, please see the FRAGTE1 study (Zhou et al., 2020).

**Query**	**Query name**	**Reference**	**Reference name**	**P1**	**P2**	**LSC**	**GSC_q**	**GSC_r**	**ANI (%)**	**PSG (%)**
GCA_001313785.1	*Gordonia westfalica*	GCA_900105725.1	*Gordonia westfalica*	0.88	0.89	0.75	0.92	0.82	98.98	72.93
GCA_000698455.1	*Rhodococcus qingshengii*	GCA_001313445.1	*Rhodococcus qingshengii*	0.83	0.85	0.75	0.8	0.92	97.78	82.75

Supplementary Table 5. The indeed interspecific pairs from labeled intraspecific pairs uniquely sieved by FRAGTE1 on real genomes. P1, PCCD based on ZRFs; P2, PCCD based on z-scores of the fourfold longer fragment; LSC, length-specific cutoff in FRAGTE1. For details, please see the FRAGTE1 study (Zhou et al., 2020). This table is in a separate file.

Supplementary Table 6. The intraspecific pairs unsieved by FRAGTE2 on real genomes.

Query	Query name	Reference	Reference name	PCCD	GSC_q	GSC_r	ANI (%)	PSG (%)
GCA_001315865.1	*Methanosarcina mazei*	GCA_000970205.1	*Methanosarcina mazei*	0.85	0.96	0.98	97.87	86.09
GCA_001315905.1	*Thermococcus peptonophilus*	GCA_001592435.1	*Thermococcus peptonophilus*	0.88	0.97	0.98	98.91	99.79
GCA_001316145.1	*Vulcanisaeta distributa*	GCA_000148385.1	*Vulcanisaeta distributa*	0.88	0.96	0.98	96.41	84.26
GCA_001313725.1	*Gordonia amicalis*	GCA_000332995.1	*Gordonia amicalis*	0.89	0.97	0.97	98.95	79.54
GCA_001313785.1	*Gordonia westfalica*	GCA_900105725.1	*Gordonia westfalica*	0.89	0.97	0.91	98.98	72.93
GCA_001312045.1	*Lactobacillus satsumensis*	GCA_001435195.1	*Lactobacillus satsumensis*	0.86	0.95	0.93	99.48	99.65
GCA_001313425.1	*Rhodococcus phenolicus*	GCA_001646785.1	*Rhodococcus phenolicus*	0.89	0.97	0.95	98.9	77.77
GCA_001313465.1	*Granulicoccus phenolivorans*	GCA_000423085.1	*Granulicoccus phenolivorans*	0.89	0.97	0.95	98.59	80.71
GCA_001314125.1	*Mycobacterium aromaticivorans*	GCA_000559085.2	*Mycobacterium aromaticivorans*	0.84	0.97	0.98	98.64	81.97
GCA_000341815.1	*Rhodococcus qingshengii*	GCA_001313445.1	*Rhodococcus qingshengii*	0.87	0.96	0.96	97.9	86.99
GCA_000698455.1	*Rhodococcus qingshengii*	GCA_001313445.1	*Rhodococcus qingshengii*	0.87	0.91	0.96	97.78	82.75
GCA_001623435.1	*Rhodococcus qingshengii*	GCA_001313445.1	*Rhodococcus qingshengii*	0.87	0.98	0.96	97.98	87.75
GCA_001646745.1	*Rhodococcus qingshengii*	GCA_001313445.1	*Rhodococcus qingshengii*	0.87	0.95	0.96	99.08	98.94
GCA_001662505.1	*Rhodococcus qingshengii*	GCA_001313445.1	*Rhodococcus qingshengii*	0.87	0.98	0.96	97.99	85.44

Supplementary Table 7. The intraspecific pairs unsieved by FRAGTE2 but with other intraspeciesfic references sieved by FRAGTE2 on real genomes. PCCD, Pearson correlation coefficient distance; GSC_q, genome-specific cutoff for query; GSC_r, genome-specific cutoff for query; ANI, average nucleotide identity; PSG, percentage of shared genome; red, the intraspecific pair unsived by FRAGTE2.

Query	Query name	Reference	Reference name	PCCD	GSC_q	GSC_r	ANI (%)	PSG (%)
GCA_001646745.1	*Rhodococcus qingshengii*	GCA_001313445.1	*Rhodococcus qingshengii*	0.87	0.95	0.96	99.08	98.94
GCA_001646745.1	*Rhodococcus qingshengii*	GCA_001311605.1	*Rhodococcus baikonurensis*	1	0.95	0.96	98.36	87.01
GCA_001646745.1	*Rhodococcus qingshengii*	GCA_001456965.1	*Rhodococcus enclensis*	1	0.95	0.95	98.5	84.45
GCA_001646745.1	*Rhodococcus qingshengii*	GCA_001552595.1	*Rhodococcus erythropolis*	1	0.95	0.96	95.22	85.73
GCA_001623435.1	*Rhodococcus qingshengii*	GCA_001311605.1	*Rhodococcus baikonurensis*	1	0.98	0.96	98.61	89.24
GCA_001623435.1	*Rhodococcus qingshengii*	GCA_001313445.1	*Rhodococcus qingshengii*	0.87	0.98	0.96	97.98	87.75
GCA_001623435.1	*Rhodococcus qingshengii*	GCA_001456965.1	*Rhodococcus enclensis*	1	0.98	0.95	98.59	84.88
GCA_001623435.1	*Rhodococcus qingshengii*	GCA_001552595.1	*Rhodococcus erythropolis*	1	0.98	0.96	95.26	87.82
GCA_000341815.1	*Rhodococcus qingshengii*	GCA_001311605.1	*Rhodococcus baikonurensis*	1	0.96	0.96	98.49	87.94
GCA_000341815.1	*Rhodococcus qingshengii*	GCA_001456965.1	*Rhodococcus enclensis*	1	0.96	0.95	98.65	86.45
GCA_000341815.1	*Rhodococcus qingshengii*	GCA_001313445.1	*Rhodococcus qingshengii*	0.87	0.96	0.96	97.9	86.99
GCA_000341815.1	*Rhodococcus qingshengii*	GCA_001552595.1	*Rhodococcus erythropolis*	1	0.96	0.96	95.31	88.47
GCA_000698455.1	*Rhodococcus qingshengii*	GCA_001456965.1	*Rhodococcus enclensis*	1	0.91	0.95	98.58	85.05
GCA_000698455.1	*Rhodococcus qingshengii*	GCA_001311605.1	*Rhodococcus baikonurensis*	1	0.91	0.96	98.43	83.47
GCA_000698455.1	*Rhodococcus qingshengii*	GCA_001313445.1	*Rhodococcus qingshengii*	0.87	0.91	0.96	97.78	82.75
GCA_000698455.1	*Rhodococcus qingshengii*	GCA_001552595.1	*Rhodococcus erythropolis*	1	0.91	0.96	95.34	83.62
GCA_001662505.1	*Rhodococcus qingshengii*	GCA_001311605.1	*Rhodococcus baikonurensis*	1	0.98	0.96	98.56	87.44
GCA_001662505.1	*Rhodococcus qingshengii*	GCA_001313445.1	*Rhodococcus qingshengii*	0.87	0.98	0.96	97.99	85.44
GCA_001662505.1	*Rhodococcus qingshengii*	GCA_001552595.1	*Rhodococcus erythropolis*	1	0.98	0.96	95.28	87.04
GCA_001662505.1	*Rhodococcus qingshengii*	GCA_001456965.1	*Rhodococcus enclensis*	1	0.98	0.95	98.55	83.45

Supplementary Table 8. The intraspecific pairs unsieved by FRAGTE1 on real genomes. P1, PCCD based on ZRFs; P2, PCCD based on z-scores of the fourfold longer fragment; LSC, length-specific cutoff in FRAGTE1. For details, please see the FRAGTE1 study (Zhou et al., 2020).

Query	Query name	Reference	Reference name	P1	P2	LSC	GSC_q	GSC_r	ANI (%)	PSG (%)
GCA_001315865.1	*Methanosarcina mazei*	GCA_000970205.1	*Methanosarcina mazei*	0.83	0.83	0.75	0.92	0.92	97.87	86.09
GCA_001315905.1	*Thermococcus peptonophilus*	GCA_001592435.1	*Thermococcus peptonophilus*	0.84	0.85	0.75	0.92	0.92	98.91	99.79
GCA_001316145.1	*Vulcanisaeta distributa*	GCA_000148385.1	*Vulcanisaeta distributa*	0.84	0.85	0.75	0.92	0.92	96.41	84.26
GCA_000319595.1	*Cronobacter sakazakii*	GCA_000982825.1	*Cronobacter sakazakii*	0.9	0.91	0.75	0.92	0.92	96.2	82.23
GCA_001313725.1	*Gordonia amicalis*	GCA_000332995.1	*Gordonia amicalis*	0.87	0.89	0.75	0.92	0.92	98.95	79.54
GCA_000472165.1	*Ochrobactrum intermedium*	GCA_000182645.1	*Ochrobactrum intermedium*	0.89	0.89	0.75	0.92	0.92	99.22	95.32
GCA_001312045.1	*Lactobacillus satsumensis*	GCA_001435195.1	*Lactobacillus satsumensis*	0.83	0.85	0.75	0.92	0.9	99.48	99.65
GCA_001313425.1	*Rhodococcus phenolicus*	GCA_001646785.1	*Rhodococcus phenolicus*	0.89	0.89	0.75	0.92	0.92	98.9	77.77
GCA_001313465.1	*Granulicoccus phenolivorans*	GCA_000423085.1	*Granulicoccus phenolivorans*	0.88	0.89	0.75	0.92	0.92	98.59	80.71
GCA_001312025.1	*Lysinibacillus boronitolerans*	GCA_000772935.1	*Lysinibacillus boronitolerans*	0.88	0.91	0.75	0.92	0.92	99.58	99.65
GCA_001314125.1	*Mycobacterium aromaticivorans*	GCA_000559085.2	*Mycobacterium aromaticivorans*	0.84	0.84	0.75	0.92	0.92	98.64	81.97
GCA_000341815.1	*Rhodococcus qingshengii*	GCA_001313445.1	*Rhodococcus qingshengii*	0.84	0.85	0.75	0.92	0.92	97.9	86.99
GCA_001623435.1	*Rhodococcus qingshengii*	GCA_001313445.1	*Rhodococcus qingshengii*	0.84	0.85	0.75	0.92	0.92	97.98	87.75
GCA_001646745.1	*Rhodococcus qingshengii*	GCA_001313445.1	*Rhodococcus qingshengii*	0.84	0.85	0.75	0.92	0.92	99.08	98.94
GCA_001662505.1	*Rhodococcus qingshengii*	GCA_001313445.1	*Rhodococcus qingshengii*	0.86	0.86	0.75	0.92	0.92	97.99	85.44
GCA_001436555.1	*Lactobacillus senioris*	GCA_001312065.1	*Lactobacillus senioris*	0.9	0.9	0.75	0.92	0.92	99.47	99.97

Supplementary Table 9. The intraspecific pairs unsieved by FRAGTE1 with other intraspeciesfic references sieved by FRAGTE1 on real genomes. P1, PCCD based on ZRFs; P2, PCCD based on z-scores of the fourfold longer fragment; LSC, length-specific cutoff in FRAGTE1; red, the intraspecific pair unsived by FRAGTE1. For details, please see the FRAGTE1 study (Zhou et al., 2020).

Query	Query name	Reference	Reference name	P1	P2	LSC	GSC_q	GSC_r	ANI (%)	PSG (%)
GCA_001646745.1	*Rhodococcus qingshengii*	GCA_001313445.1	*Rhodococcus qingshengii*	0.84	0.85	0.75	0.92	0.92	99.08	98.94
GCA_001646745.1	*Rhodococcus qingshengii*	GCA_001311605.1	*Rhodococcus baikonurensis*	0.98	0.99	0.75	0.92	0.92	98.33	87.15
GCA_001646745.1	*Rhodococcus qingshengii*	GCA_001456965.1	*Rhodococcus enclensis*	0.98	0.99	0.75	0.92	0.92	98.47	84.68
GCA_001646745.1	*Rhodococcus qingshengii*	GCA_001552595.1	*Rhodococcus erythropolis*	0.97	0.99	0.75	0.92	0.92	95.2	85.96
GCA_001623435.1	*Rhodococcus qingshengii*	GCA_001311605.1	*Rhodococcus baikonurensis*	0.98	0.99	0.75	0.92	0.92	98.58	89.33
GCA_001623435.1	*Rhodococcus qingshengii*	GCA_001313445.1	*Rhodococcus qingshengii*	0.84	0.85	0.75	0.92	0.92	97.98	87.75
GCA_001623435.1	*Rhodococcus qingshengii*	GCA_001456965.1	*Rhodococcus enclensis*	0.98	0.99	0.75	0.92	0.92	98.57	85.06
GCA_001623435.1	*Rhodococcus qingshengii*	GCA_001552595.1	*Rhodococcus erythropolis*	0.97	0.99	0.75	0.92	0.92	95.25	87.92
GCA_000341815.1	*Rhodococcus qingshengii*	GCA_001311605.1	*Rhodococcus baikonurensis*	0.97	0.99	0.75	0.92	0.92	98.44	88.15
GCA_000341815.1	*Rhodococcus qingshengii*	GCA_001456965.1	*Rhodococcus enclensis*	0.97	0.99	0.75	0.92	0.92	98.62	86.61
GCA_000341815.1	*Rhodococcus qingshengii*	GCA_001313445.1	*Rhodococcus qingshengii*	0.84	0.85	0.75	0.92	0.92	97.9	86.99
GCA_000341815.1	*Rhodococcus qingshengii*	GCA_001552595.1	*Rhodococcus erythropolis*	0.97	0.99	0.75	0.92	0.92	95.3	88.65
GCA_001662505.1	*Rhodococcus qingshengii*	GCA_001311605.1	*Rhodococcus baikonurensis*	0.97	0.99	0.75	0.92	0.92	98.52	87.56
GCA_001662505.1	*Rhodococcus qingshengii*	GCA_001313445.1	*Rhodococcus qingshengii*	0.86	0.86	0.75	0.92	0.92	97.99	85.44
GCA_001662505.1	*Rhodococcus qingshengii*	GCA_001552595.1	*Rhodococcus erythropolis*	0.98	0.99	0.75	0.92	0.92	95.26	87.17
GCA_001662505.1	*Rhodococcus qingshengii*	GCA_001456965.1	*Rhodococcus enclensis*	0.98	1	0.75	0.92	0.92	98.53	83.6

Supplementary Table 10. The exceptions unsieved by FRAGTE2 with more other probable targets (intraspeciesfic references) sieved by FRAGTE2 on MAGs. PCCD, Pearson correlation coefficient distance; GSC_q, genome-specific cutoff for query; GSC_r, genome-specific cutoff for query; ANI, average nucleotide identity; PSG, percentage of shared genome; gray, the exception unsived by FRAGTE2.

Query	Query name	Reference	Reference name	PCCD	GSC_q	GSC_r	ANI (%)	PSG (%)
GCA_900282665.1	human gut metagenome	GCA_900284055.1	human gut metagenome	0.99	0.91	0.91	99.91	91.24
GCA_900282665.1	human gut metagenome	GCA_900282615.1	human gut metagenome	0.96	0.91	0.91	99.95	76.74
GCA_900282665.1	human gut metagenome	GCA_900285595.1	human gut metagenome	0.86	0.91	0.91	99.9	70.59
GCA_900285595.1	human gut metagenome	GCA_900284685.1	human gut metagenome	0.99	0.91	0.91	99.74	84.61
GCA_900285595.1	human gut metagenome	GCA_900284045.1	human gut metagenome	0.99	0.91	0.91	99.94	83.02
GCA_900285595.1	human gut metagenome	GCA_900284705.1	human gut metagenome	0.98	0.91	0.91	99.92	79.8
GCA_900285595.1	human gut metagenome	GCA_900282665.1	human gut metagenome	0.86	0.91	0.91	99.9	70.59

Supplementary Table 11. Sequencers and for truely intraspecific pairs unsieved by FRAGTE2. Red, the genome sequenced by Ion Torrent Personal Genome Machine as evidenced from NCBI. Tetra1, the first differentest tetranucleotide; Tetra2, the second differentest tetranucleotide; Tetra1, the third differentest tetranucleotide.

**Query**	**Query name**	**Reference**	**Reference name**	**Tetra1**	**Tetra2**	**Tetra3**
GCA_001315865.1	*Methanosarcina mazei*	GCA_000970205.1	*Methanosarcina mazei*	GGGG	GGCC	CAGG
GCA_001315905.1	*Thermococcus peptonophilus*	GCA_001592435.1	*Thermococcus peptonophilus*	GGGG	AGGG	TCCC
GCA_001316145.1	*Vulcanisaeta distributa*	GCA_000148385.1	*Vulcanisaeta distributa*	GGGG	AGGG	ACCC
GCA_001313725.1	*Gordonia amicalis*	GCA_000332995.1	*Gordonia amicalis*	GGGG	CGGG	GGGC
GCA_001313785.1	*Gordonia westfalica*	GCA_900105725.1	*Gordonia westfalica*	GGGG	GGTG	GCCG
GCA_001312045.1	*Lactobacillus satsumensis*	GCA_001435195.1	*Lactobacillus satsumensis*	GGGG	TGGG	TCCC
GCA_001313425.1	*Rhodococcus phenolicus*	GCA_001646785.1	*Rhodococcus phenolicus*	GGGG	CGGG	GGGC
GCA_001313465.1	*Granulicoccus phenolivorans*	GCA_000423085.1	*Granulicoccus phenolivorans*	GGGG	CGGG	GGGC
GCA_001314125.1	*Mycobacterium aromaticivorans*	GCA_000559085.2	*Mycobacterium aromaticivorans*	GGTG	GGGG	GACG
GCA_000341815.1	*Rhodococcus qingshengii*	GCA_001313445.1	*Rhodococcus qingshengii*	GGGG	CGGG	GGGC
GCA_000698455.1	*Rhodococcus qingshengii*	GCA_001313445.1	*Rhodococcus qingshengii*	GGGG	CGGG	GGTG
GCA_001623435.1	*Rhodococcus qingshengii*	GCA_001313445.1	*Rhodococcus qingshengii*	GGTG	GACG	GGGG
GCA_001646745.1	*Rhodococcus qingshengii*	GCA_001313445.1	*Rhodococcus qingshengii*	GGGG	CGGG	GGGC
GCA_001662505.1	*Rhodococcus qingshengii*	GCA_001313445.1	*Rhodococcus qingshengii*	GGTG	GACG	GGGG

Reference

Zhou, Y., Zheng, J., Wu, Y., Zhang, W., and Jin, J. (2020). A completeness-independent method for pre-selection of closely related genomes for species delineation in prokaryotes. *BMC Genomics* 21**,** 183.
